# PROMIS in routine clinical care: a scoping review of implementation and use

**DOI:** 10.1186/s41687-026-01161-7

**Published:** 2026-07-29

**Authors:** Kyle Nolla, Merell Turner, George J. Greene, Amy Sparling, Richard Gershon, Michelle Langer

**Affiliations:** 1 Department of Psychology, Morgan State University, Baltimore, USA; 2 Department of Medical Social Sciences, Northwestern University, Evanston, USA; 3 KPMG (United States), Chicago, USA

**Keywords:** PROMIS, Patient-reported outcomes, Implementation, Clinical care, Narrative review

## Abstract

**Purpose:**

The Patient Reported Outcomes Measurement Information System **(**PROMIS®) is a family of patient-reported outcome measures used in research and clinical practice, but little is known about its use in clinical care. This scoping review identified studies of PROMIS in routine clinical care to understand who is using PROMIS in routine clinical care and how.

**Methods:**

We conducted a structured search of published literature in PubMed, Scopus, Embase, and *Journal of Patient-Reported Outcomes*, and hand-searched conference proceedings from the PROMIS Health Organization, International Society for Quality of Life Research, and HealthMeasures User Conferences. Studies were eligible for inclusion if they described routine use of PROMIS in a clinical setting to inform patient treatment, including studies that described implementation of PROMIS and studies that retroactively analyzed PROMIS scores collected as part of routine care. We coded 13 variables regarding study type, data collection setting, and PROMIS administration methods.

**Results:**

Our search identified 428 studies inclusive of 167 published papers and 261 conference abstracts; of these, 31% described implementing PROMIS into routine clinical care and 69% presented data resulting from use of PROMIS in routine clinical care. These studies were drawn from 77 unique institutions largely within the United States (81%) and among adult populations (59%). Academic medical centers were the most common type of institution (69%) producing 92% of identified studies. Over 20 specialties have used PROMIS in routine clinical care, with many studies from orthopedics (49%), oncology (8%), and rheumatology (6%). The most popular PROMIS domains utilized in this context included physical function, pain interference, depression, anxiety, and fatigue. All modes of administration were well-utilized (computer adaptive tests at 52%, profiles at 45%, static forms at 44%). In 69% of institutions, PROMIS is administered alongside other PROs. PROMIS is often integrated with the electronic health record (44%) and administered in clinic waiting rooms (58%).

**Conclusions:**

Adoption of PROMIS seems most robust in academic medical centers, outpatient settings, and specialties with established cultures of patient-reported outcome collection, such as orthopedics. More outreach is needed to understand how PROMIS functions in smaller clinics that do not publish or present in academic spaces.

**Supplementary Information:**

The online version contains supplementary material available at https://doi.org/10.1186/s41687-026-01161-7.

## Abstract (plain language summary)

PROMIS is a family of patient-reported outcome measures in survey format that are used to assess patient experiences of symptoms (e.g. fatigue, depression). Across conditions PROMIS is applicable to adults and children where measures evaluate physical, mental, and social functioning symptomatology, along with overall perceptions of health. Over the last twenty years, PROMIS has been adopted into clinical settings for use in routine patient care. This study reviews published research and unpublished conference abstracts to understand the types of places using PROMIS in routine care and how they do so. A wide variety of medical specialties use PROMIS to assess numerous symptoms, using several different formats. Overall, the study concludes that adoption of PROMIS seems most robust in academic medical centers, outpatient settings, and specialties with established cultures of patient-reported outcome collection, such as orthopedics. Additional research is needed to understand the factors that might improve adoption in smaller clinics.

**Supplementary Information:**

The online version contains supplementary material available at https://doi.org/10.1186/s41687-026-01161-7.

## Introduction

PROMIS is a set of person-centered measures that evaluates and monitors physical, mental, and social health in adults and children. PROMIS began in 2004 as a NIH roadmap initiative to develop and validate patient-reported outcome measures (PROMs) using state-of-the-science methods to transform how patients’ symptoms, functional status, and health-related quality of life are measured [1]. Initially, the primary focus of PROMIS-based research was on development and construct validation [2–6]. This earlier work established the psychometric and clinical validity of PROMIS, which in turn supported the implementation of PROMIS into clinical care for the purposes of patient monitoring and treatment [7]. This review focuses on the use of PROMIS in clinical care.

PROMIS offers specific advantages for clinical uses above and beyond existing PROMs. With PROMIS, PROM content can be administered as fixed-length forms or can be tailored to the patient using computer adaptive testing (CAT). PROMIS CATs display only the maximally informative items to patients, reducing the number of questions they need to answer while maintaining measurement precision and eliminating floor and ceiling effects [4]. Clinical precision can often be obtained in as little as 4 items. Similarly, PROMIS static forms are constructed to be maximally informative for the greatest number of patients and can cover single health domains (e.g., depression) or multiple domains [8]. PROMIS also offers profiles which aggregate a set of domain specific static forms into a single administration (e.g., PROMIS-29). CAT and static forms are derived from the same item bank making the scores directly comparable without the need for score conversion. Whether a CAT or a static form is used, many PROMIS scores are interpreted against severity thresholds of *within normal limits*, *mild*,* moderate*, or *severe* ranges and percentiles relative to the general population can be computed to aid in clinical decision-making [9].

Key to implementing PROMIS into routine care is integration into the electronic medical record [10], ideally using software that allows for CAT administration such as the PROMIS CAT application within the Epic electronic health record (Epic Systems Corporation, Verona, WI). However, research has revealed technical, logistical, social, and organizational challenges of integrating any PROMs into clinical care, let alone CATs which require digital administration. These challenges include uncertain clinical benefits, task overload, clinical workflow disruption, and lack of longitudinal data display [11–15]. Little research has examined whether these general implementation challenges apply to PROMIS CATs specifically; how different specialties and settings are addressing these challenges for their distinct needs; or what patterns in PROMIS adoption might reveal about facilitators and barriers to successful implementation.

To understand how PROMIS is used outside of controlled research contexts, we conducted a scoping review to characterize PROMIS use in clinical practice. Specifically, this review aims to identify who is using PROMIS in routine clinical care institution types, specialties, settings, patient populations and how it is being implemented – symptoms measured, administration formats, and integration methods. By synthesizing across all eligible published studies and conference proceedings, we can identify patterns in adoption, gaps in reporting, and opportunities for broader and more equitable dissemination of PROMIS.

## Methods

To identify literature on implementation of PROMIS measures in clinical practice, a medical librarian searched PubMed, Scopus, Embase, and the *Journal of Patient-Reported Outcomes* in August 2025. Keywords and subject headings included: *PROMIS*,* implementation*,* integration*,* uptake*,* impact*,* system wide*,* program development*, and *hospital administration*, and the search was additionally guided by a list of universities known to be PROMIS clinical users. Detailed search queries for PubMed and Embase are included in supplementary materials (Supplementary Appendix 1). The review did not follow a pre-registered protocol. Reference checking was performed as a supplemental retrieval strategy to capture works that may have been missed by the initial database search.

Additionally, conference proceedings from the PROMIS Health Organization (PHO) 2015–2024 conferences, ISOQOL 2010–2024 conferences, and the HealthMeasures User Conferences in 2017 and 2019 were hand-searched. Inclusion of this gray literature ensures greater representation of non-academic PROMIS users, who often attend conferences for continuing education and networking, and do not necessarily prioritize academic publication.

Studies were eligible if they described routine use of PROMIS in a clinical setting to inform a clinician about a patient’s health for purposes of treatment. Ultimately, the studies meeting these criteria fell into two categories. The first category was *implementation studies*, or studies describing the process of implementing PROMIS into routine clinical care, including studies evaluating the appropriateness and feasibility of PROMIS for long-term, routine clinical use. The second category was *routine care results*, or studies that retroactively review and draw conclusions from PROMIS scores that were collected as part of routine care. Studies were not eligible if they described clinical trials or other short-term interventions, research registries where the treating clinician did not review PROMIS scores, or other studies where PROMIS scores were not reviewed by a clinician to inform treatment. Eligibility screening was performed by two authors independently.

Each paper was coded for twelve additional variables of interest. First, seven variables described the data collection setting, including (1) the institution or institutions where PROMIS was implemented, (2) the type of institution (academic medical centers or non-academic), (3) the country, (4) the medical specialties (e.g., oncology), (5) the clinic setting (e.g., outpatient), (6) the population (adult, pediatric, or both), and (7) the years when data were collected. Second, five variables described administration of PROMIS measures, including (1) whether PROMIS was integrated with the electronic health record, (2) the location PROMIS measures were administered (e.g., clinic waiting room), (3) the PROMIS domains measure (e.g., physical function, depression, global health), (4) the format of PROMIS measures (profiles, static forms, CATs), and (5) whether other PROMs were administered alongside PROMIS. Two authors performed coding. One author developed and applied the coding strategy to most studies, then trained another author to code. The second coder re-analyzed several previous studies, which were checked by the original coder to ensure consistency and clarity of instructions; then, the second coder completed the remaining studies.

To understand how PROMIS functions at the institutional level, articles published from the same institutions were grouped together for some analyses. If information conflicted – for example, one institution’s pediatric clinics used a different set of PROMIS measures than their adult oncology department – codes were combined so that both departments were represented within the same institution (e.g., both the Pediatric Profile and PROMIS Global Health were included in the list of measures used by the institution). Synthesis of codes involved basic counts and frequencies for each code at the study level or institution level, as appropriate for each code.

## Results

**Fig. 1:**
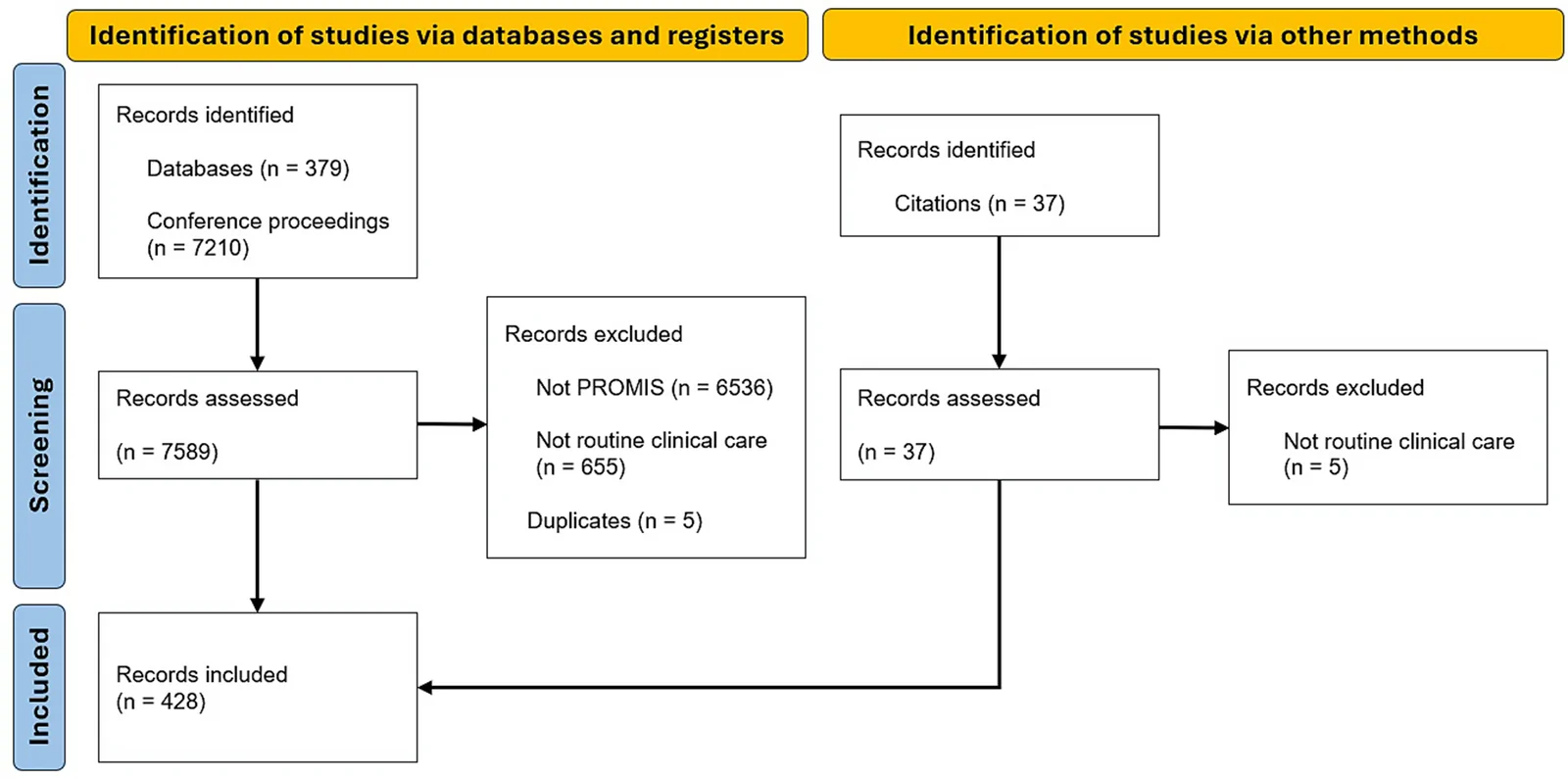
A flow diagram of record identification and eligibility screening

The literature search identified 379 published papers and 7210 conference abstracts for a total of 7589 records that were assessed for eligibility. Reports were first excluded if they did not involve PROMIS (*n* = 6536), then if they did not describe PROMIS in routine clinical care (*n* = 655). Several conference abstracts were later published as manuscripts, thus were excluded as duplicates (*n* = 5). During coding, 37 additional published papers were identified from citations, and 32 of these were eligible for inclusion. In total, 428 studies including 167 (39%) published articles and 261 (61%) conference abstracts were reviewed. (See Fig. 1 & Supplementary Appendix 2.)

31% (132) of studies described implementation of PROMIS into routine clinical care while 69% (296) reported data that had been routinely collected during clinical care.

### Data collection setting

Across all eligible studies, 77 unique institutions inclusive of over 30 different specialties were identified (see Supplementary Table 1). Academic medical centers were the most common type of institution at 69% (53); non-academic institutions included community-based, government, military, and privately-run institutions. As expected, academic medical centers accounted for most studies identified in our search (393 of 428 studies or 92%).

Most institutions (62; 81%) were located within the United States. Other countries included the Netherlands (5), Germany (3); Canada (2); Australia (2); the United Kingdom (1); Bolivia (1); and Hungary (1).

The most common specialty to publish studies on PROMIS in clinical care was orthopedics, representing 49% (211) of studies. Other common specialties included oncology with 8.4% (36) of studies and rheumatology with 6.1% (26) of studies. The remaining 36% of studies included over 20 other specialties including dermatology, neurology, general surgery, and behavioral health.

Studies conducted in outpatient settings were overwhelmingly the most common (96%), with the remaining settings including inpatient, both inpatient and outpatient, and home health.

Data from exclusively adult participants was used in 59% (253) of studies. 8.2% (35) reported on pediatric populations. 2.6% (11) reported on both pediatric and adult samples. The remaining studies (129; 30%) did not specify the age range of their samples.

**Fig. 2:**
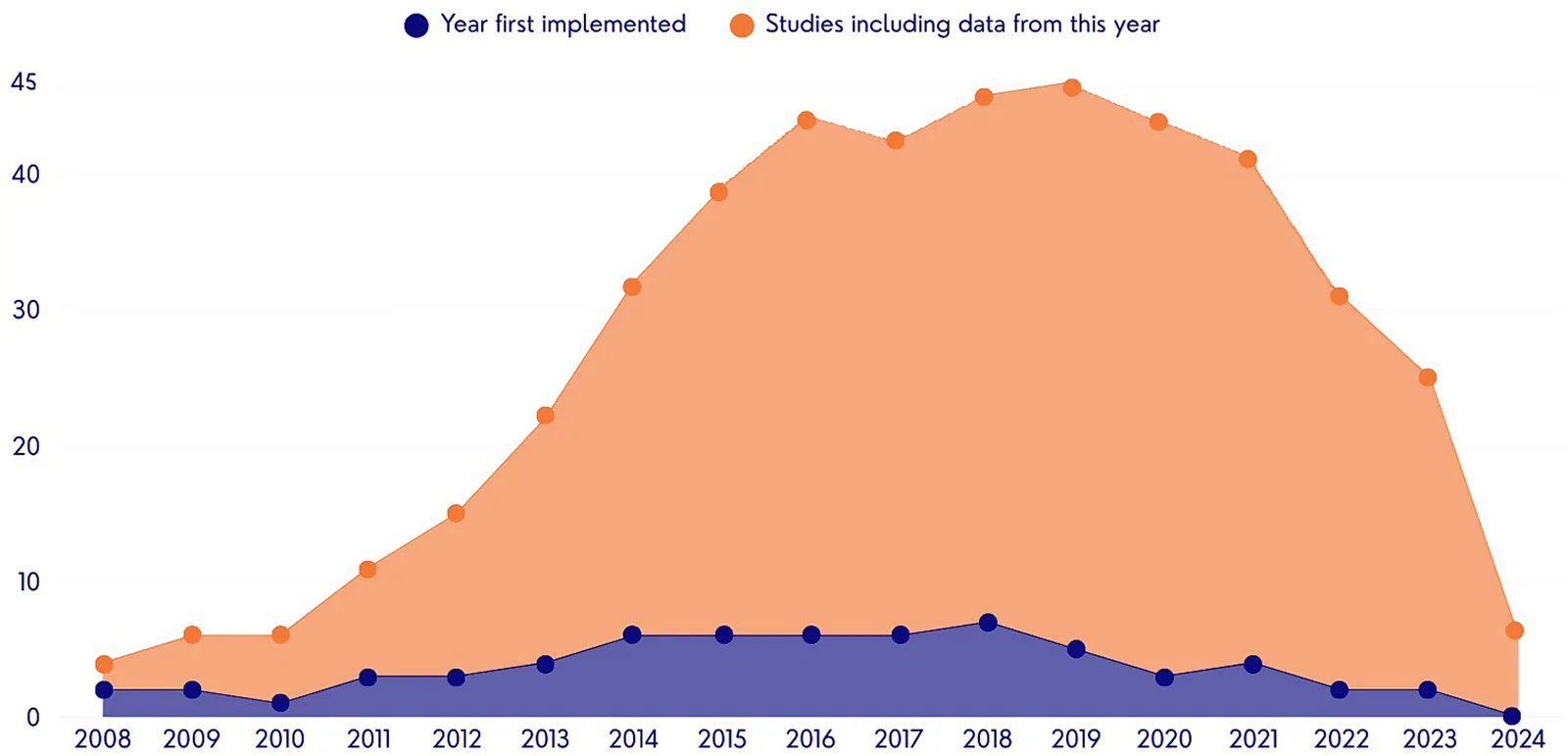
PROMIS has been implemented yearly at new institutions since 2008, with continued usage consistent with cumulative implementation rates

Institutions described PROMIS as being first implemented in all years ranging from 2008 to 2023 (see Fig. 2), with the most institutions (*n* = 7) reporting first implementation in 2018. Studies included data spanning from 2008 to 2024, with the number of studies incorporating data from a given year peaking in 2019 (*n* = 45). The subsequent decline in both implementation counts and studies-with-data from 2022 to 2024 likely reflects publication lag rather than reduced PROMIS use.

### Administration of PROMIS

**Fig. 3:**
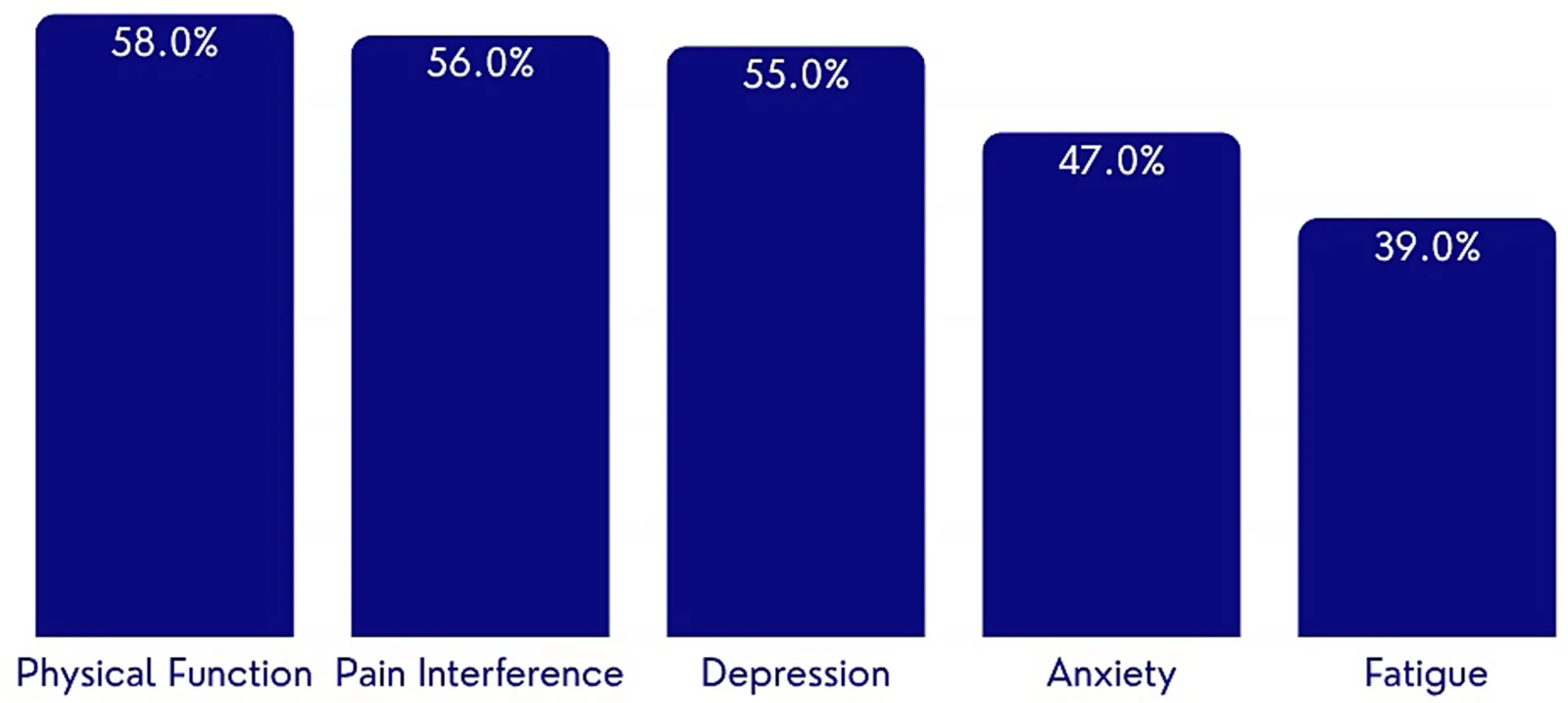
The five most popular PROMIS domains and the proportion of institutions from the review using each domain

The 77 institutions collected 30 unique PROMIS domains. The most popular domains included physical function (given at 45 or 58% of institutions) and pain interference (43; 56%), consistent with the popularity of PROMIS in orthopedics. Other popular domains include depression (42; 55%), anxiety (36; 47%), and fatigue (30; 39%; see Fig. 3). 31% of institutions (24) used PROMIS Global Health, which evaluates global mental and physical health through a 10-item survey worded to provide a broad overview of global health (e.g., *In general*,* would you say your health is…?*).

PROMIS measures were administered in several different formats. About a third of institutions (26; 34%) used more than one format (e.g., PROMIS Global Health and a physical function CAT), thus totals reported here add up to greater than 100%. Across institutions, PROMIS CATs were the most popular (40; 52%) followed by profiles (35; 45%) and static forms (34; 44%). A logistic regression testing whether institution type (academic or non-academic) predicted any use of CATs was significant such that non-academic institutions had lower odds of using CATs that non-academic institutions (OR, 0.35; 95% CI, 0.12–0.95; *P* = 0.04).

Often, PROMIS measures were administered alongside PROMs not from the PROMIS family of measures (53; 69%). Disease specific PROMs typically measured more specialized information than collected by PROMIS, such as the KOOS-JR [16] administered for knee-specific physical function to patients preparing for knee replacement surgery.

In line with recommended practice, PROMIS was automatically integrated with the electronic health record (EHR) at 44% (34) of institutions. PROMIS was manually paired with the EHR – such as staff typing PROMIS scores into patient notes – in much fewer cases (4; 5.2%). 12 institutions (12; 16%) reported that PROMIS was *not* integrated with the EHR. 32% (25) of institutions did not report whether their PROMIS measures were EHR-integrated.

Institutions and departments differed in where they administered PROMIS. The most common location for PROMIS administration was the clinic waiting room (45; 58%), followed by online before the appointment (36; 47%). About a quarter of institutions offered PROMIS both online before the appointment and in the waiting room if the patient did not complete it online before arriving (22; 29%; included in counts above). A few institutions administered PROMIS elsewhere in the clinic such as in the examination room (4; 5%), and one home health study administered PROMIS via tablet during their home health appointment. Finally, for 21% (16) of institutions, no studies described the administration location.

## Discussion

This review demonstrates the versatility of PROMIS as a clinical tool, and the patterns of its adoption reveal important insights about implementation feasibility and barriers. PROMIS offers flexibility and variety for measurement of patient-reported outcomes, and clinical users adopt PROMIS as appropriate to suit many different clinical contexts. PROMIS has gained strongest traction in settings with robust research infrastructure, particularly academic medical centers and orthopedic departments, and in clinical contexts with established workflows for patient-reported outcome collection [e.g., 10, 12]. For digital delivery of CATs, academic institutions often have dedicated informatics departments, research personnel, familiarity with PROMs, and institutional support for innovation that non-academic settings may lack, suggesting that substantial resources are necessary for successful implementation [14, 15] – although resources alone are not always sufficient for widespread patient uptake [17]. Fewer than half of the institutions identified in the review had PROMIS fully integrated with the EHR. The recent integration of PROMIS into widely used electronic health record systems like Epic lowers many technical barriers, potentially accelerating adoption in non-academic settings. Still, non-academic institutions may require targeted support in areas such as workflow redesign, clinician and staff training, and demonstrating clinical value to leadership [14, 15].

The concentration of studies in orthopedics may reflect that specialty’s long-standing culture of using patient-reported outcomes to track functional status and surgical outcomes. Orthopedic surgeons have a clear use case for physical function measurement and a history of using legacy measures prior to PROMIS, making adoption more intuitive [18, 19]. At the institution level, the clustering of orthopedics studies within specific institutions suggests that individual clinician-research champions may drive adoptions of PROMIS and dissemination of resulting research. Beyond orthopedics, some specialties such as oncology [20] and rheumatology [21] have demonstrated meaningful uptake, suggesting that PROMIS can address diverse clinical needs when appropriately tailored. However, specialties without established PRO workflows – including inpatient and home health settings which were greatly underrepresented in studies identified by this review – may require additional support in identifying relevant symptom domains, integrating PRO review into clinical encounters, and demonstrating value for their specific patient populations and clinical decision-making processes.

The variety of PROMIS formats (e.g., CATs, static forms, profiles), symptom domains (e.g., depression, fatigue), and administration methods (e.g., waiting room tablets, online patient portals) demonstrates the measure system’s adaptability to different clinical contexts and technological capabilities. Institutions are not implementing PROMIS in a one-size-fits-all manner; rather, they are selecting formats, domains, and administration methods that align with their clinical needs, patient populations, and available infrastructure. The frequent co-administration of PROMIS with legacy measures further illustrates this pragmatic approach. Institutions using multiple administration modes, offering both online and in-clinic options, may achieve higher completion rates and more representative data collection by avoiding the digital divide that emerges due to population-level differences in online patient portal use [22]. These patterns suggest that successful implementation requires flexibility and that PROMIS functions most effectively as part of a broader assessment strategy reflecting clinician and patient needs.

The steady growth in PROMIS implementation over time suggests the measure system is moving beyond early adopters toward broader acceptance. International uptake, though still modest compared to U.S. implementation, demonstrates that PROMIS can be adapted across healthcare systems, languages, and cultures. Still, based on the gaps identified in this review, important priorities for future dissemination efforts include understanding PROMIS adoption in non-academic settings, expanding beyond orthopedics into specialties without established PRO workflows, addressing the technical and workflow challenges of EHR integration, and ensuring equitable access across diverse patient populations.

### Limitations

Several limitations should be considered when interpreting these findings. First, our reliance on published literature and conference proceedings overrepresents academic medical centers, as these institutions have greater resources dedicated to research dissemination. Community hospitals, private practices, and other non-academic settings using PROMIS may be underrepresented in our sample, meaning the true breadth of implementation may be broader than captured here. Second, this scoping review is inherently less thorough than a systematic review with preregistered methods and risk of bias assessment; our synthesis prioritized breadth and descriptive characterization over methodological rigor in study evaluation. Third, we did not directly assess barriers and facilitators through surveys or interviews with implementers; rather, we inferred these from patterns in the literature. Fourth, our final literature search was conducted in August 2025; contributions to the literature beyond our final search date were not included in our synthesis. Finally, reporting practices varied considerably across studies, particularly in conference abstracts, resulting in substantial missing data for some variables of interest. The most notable missing variables for conference abstracts was whether PROMIS scores were integrated into EHR, PROMIS administration location, and the population or sample that PROMIS was being used for in the study.

## Conclusions

This scoping review provides an overview of how PROMIS has been implemented in routine care across institutions, specialties, and settings. These findings demonstrate that PROMIS functions as a flexible clinical tool for patient monitoring and outcomes assessment, adaptable to local clinical workflows and specialized patient populations. Adoption seems most robust in academic medical centers, outpatient settings, and specialties with established cultures of patient-reported outcome collection, such as orthopedics. As EHR integration continues to improve and the evidence supporting the clinical utility of PROs grows, PROMIS is well-positioned to improve data-informed clinical care across a wide range of healthcare settings. 

## Supplementary Information

Below is the link to the electronic supplementary material.

Supplementary Material 1

Supplementary Material 2

Supplementary Material 3

## Data Availability

The primary data analyzed in this study available in the Supplementary Materials.
