# Supplementary material for: PROMIS in routine clinical care: a scoping review of implementation and use
Source: J Patient Rep Outcomes. 2026 Jul 29;10:196. doi: 10.1186/s41687-026-01161-7 (PMC13696663; doi:10.1186/s41687-026-01161-7)
Supplement: Supplementary file 1 [file 41687_2026_1161_MOESM2_ESM.docx]

**Supplementary Appendix 1.** Detailed Search Strategy.

**Embase**

| **No.** | **Query** |
| --- | --- |
| 1 | 'patient reported outcomes measurement information system'/exp OR promis:ti OR 'patient reported outcomes measurement information system':ti |
| 2 | 'program evaluation'/mj/exp OR 'evaluation study'/mj/exp OR usability:ti OR usable:ti OR useful*:ti OR practical*:ti OR applicab*:ti OR reliab*:ti OR acceptab*:ti OR feasib*:ti OR experience*:ti OR utiliz*:ti OR utility:ti OR adopt*:ti OR implement*:ti OR impact:ti OR 'clinical practice':ti OR integration:ti OR uptake:ti OR 'setting*':ti OR 'real world':ti OR 'clinical application*':ti OR 'routine*':ti OR 'clinical care':ti OR clinic:ti OR clinics:ti OR center:ti OR centers:ti OR hospital:ti OR hospitals:ti |
| 3 | 1 AND 2 |
| 4 | 'cedars sinai':ti,ab,kw OR 'mayo clinic':ti,ab,kw OR 'ohio state university':ti,ab,kw OR 'spectrum health':ti,ab,kw OR 'thomas jefferson university':ti,ab,kw OR 'university of iowa':ti,ab,kw OR 'weill cornell':ti,ab,kw OR 'university of pittsburgh':ti,ab,kw OR 'memorial hospital of laramie county':ti,ab,kw OR 'cheyenne regional medical center':ti,ab,kw OR 'parkview health system':ti,ab,kw OR 'kaiser foundation':ti,ab,kw OR 'childrens hospital colorado':ti,ab,kw OR 'university of texas':ti,ab,kw OR 'denver health and hospital authority':ti,ab,kw OR 'childrens medical center of dallas':ti,ab,kw OR 'duke university':ti,ab,kw OR 'university of michigan':ti,ab,kw OR 'university of chicago':ti,ab,kw OR 'west virginia university':ti,ab,kw OR 'osf':ti,ab,kw OR 'university of pennsylvania':ti,ab,kw OR 'ohiohealth':ti,ab,kw OR 'nemours foundation':ti,ab,kw OR 'mount sinai':ti,ab,kw OR 'university of miami':ti,ab,kw OR 'cleveland clini':ti,ab,kw OR 'highmark':ti,ab,kw OR 'allegheny':ti,ab,kw OR 'stichting':ti,ab,kw OR 'olvg':ti,ab,kw OR 'anne arundel':ti,ab,kw OR 'baylor college':ti,ab,kw OR 'providence st joseph':ti,ab,kw OR 'university of utah':ti,ab,kw OR 'university of rochester':ti,ab,kw OR 'hospital for special surgery':ti,ab,kw OR 'vermont':ti,ab,kw OR 'emory':ti,ab,kw OR 'new york university':ti,ab,kw OR 'parker institute':ti,ab,kw OR 'walter reed':ti,ab,kw OR 'army analytics group':ti,ab,kw OR 'university of alabama':ti,ab,kw OR 'palo alto':ti,ab,kw OR 'ucsf':ti,ab,kw OR 'johns hopkins':ti,ab,kw |
| 5 | (promis OR 'patient reported outcomes measurement information system') AND (integration:ti OR integrating:ti) AND (pros:ti OR proms:ti OR patient) AND reported AND outcome*:ti NOT cons:ti |
| 6 | (promis:ti OR 'patient reported outcomes measurement information system':ti) AND ('change management'/exp OR 'hospital management'/exp OR 'health care management'/de OR 'utilization review'/exp OR 'organization and management'/exp) |
| 7 | (promis OR 'patient reported outcomes measurement information system') AND ('change management'/exp OR 'hospital management'/exp OR 'health care management'/de OR 'utilization review'/exp OR 'organization and management'/exp) AND (pros:ti OR proms:ti OR patient) AND reported AND outcome*:ti NOT cons:ti |
| 8 | (promis OR 'patient reported outcomes measurement information system') AND 4 |
| 9 | 'clinical trial'/exp OR 'controlled trial':ti OR 'clinical trial':ti OR rct:ti OR 'clinical study'/de OR 'prospective study'/exp OR 'retrospective study'/exp |
| 10 | [1-10-2021]/sd NOT [01-06-2023]/sd [updated each time to reflect date of current search] |
| 11 | 3 OR 5 OR 6 OR 7 OR 8 |
| 12 | 10 AND 11 |
| 13 | 12 NOT 9 |

**PubMed**

(((PROMIS OR "patient reported outcomes measurement information system") AND (integration[ti] OR integrating[ti]) AND ((PROs[ti] OR PROMs[ti] OR patient reported outcome*[ti]) NOT cons[ti])) OR ((PROMIS[ti] OR "patient reported outcomes measurement information system"[ti]) AND ("Organizational Innovation"[Mesh] OR "Change Management"[Mesh] OR "Health Facility Administration"[Mesh] OR "organization and administration"[mesh])) OR ((PROMIS OR "patient reported outcomes measurement information system") AND ("Organizational Innovation"[Mesh] OR "Change Management"[Mesh] OR "Health Facility Administration"[Mesh] OR "organization and administration"[mesh]) AND ((PROs[ti] OR PROMs[ti] OR patient reported outcome*[ti]) NOT cons[ti])) OR ((PROMIS OR "patient reported outcomes measurement information system") AND ("cedars sinai"[tiab] OR "mayo clinic"[tiab] OR "ohio state university"[tiab] OR "spectrum health"[tiab] OR "thomas jefferson university"[tiab] OR "university of iowa"[tiab] OR "weill cornell"[tiab] OR "university of pittsburgh"[tiab] OR "memorial hospital of laramie county"[tiab] OR "cheyenne regional medical center"[tiab] OR "parkview health system"[tiab] OR "kaiser foundation"[tiab] OR "childrens hospital colorado"[tiab] OR "university of texas"[tiab] OR "denver health and hospital authority"[tiab] OR "childrens medical center of dallas"[tiab] OR "duke university"[tiab] OR "university of michigan"[tiab] OR "university of chicago"[tiab] OR "west virginia university"[tiab] OR "osf"[tiab] OR "university of pennsylvania"[tiab] OR "ohiohealth"[tiab] OR "nemours foundation"[tiab] OR "mount sinai"[tiab] OR "university of miami"[tiab] OR "cleveland clini"[tiab] OR "highmark"[tiab] OR "allegheny"[tiab] OR "stichting"[tiab] OR "olvg"[tiab] OR "anne arundel"[tiab] OR "baylor college"[tiab] OR "providence st joseph"[tiab] OR "university of utah"[tiab] OR "university of rochester"[tiab] OR "hospital for special surgery"[tiab] OR "vermont"[tiab] OR "emory"[tiab] OR "new york university"[tiab] OR "parker institute"[tiab] OR "walter reed"[tiab] OR "army analytics group"[tiab] OR "university of alabama"[tiab] OR "palo alto"[tiab] OR "ucsf"[tiab] OR "johns hopkins"[tiab])) OR ((promis[ti] OR "patient reported outcomes measurement information system"[ti]) AND ("program evaluation"[mesh:noexp] OR "evaluation study"[mesh] OR "utilization review"[mesh] OR usability[ti] OR usable[ti] OR useful*[ti] OR practical*[ti] OR applicab*[ti] OR reliab*[ti] OR acceptab*[ti] OR feasib*[ti] OR experience*[ti] OR utiliz*[ti] OR utility[ti] OR adopt*[ti] OR implement*[ti] OR impact[ti] OR "clinical practice"[ti] OR integration[ti] OR uptake[ti] OR "setting*"[ti] OR "real world"[ti] OR "clinical application*"[ti] OR "routine*"[ti] OR "clinical care"[ti] OR clinic[ti] OR clinics[ti] OR center[ti] OR centers[ti] OR hospital[ti] OR hospitals[ti]))) NOT ("Clinical Study" [Publication Type] OR RCT[ti] OR "clinical trial"[ti] OR "controlled trial"[ti])
