# Supplementary material for: PROMIS in routine clinical care: a scoping review of implementation and use
Source: J Patient Rep Outcomes. 2026 Jul 29;10:196. doi: 10.1186/s41687-026-01161-7 (PMC13696663; doi:10.1186/s41687-026-01161-7)
Supplement: Supplementary file 2 [file 41687_2026_1161_MOESM3_ESM.docx]

**Supplementary Appendix 2: List of cited studies.**

**Published Literature**

| # | Citation |
| --- | --- |
| 1 | Abouzeid, Cailin, et al. “Fractional CO2 Laser for Burn Scars: A Comparison of Patient-Reported Outcomes Between Those With and Without Laser Treatment.” Journal of Burn Care & Research, vol. 45, no. 6, 2024, pp. 1505–12, https://doi.org/10.1093/jbcr/irae129. |
| 2 | Adams, E., Carle, J., Da Silva, Z., Rasmussen, G., & Grudziak, J. (2023). PROMIS 29 in Emergency General Surgery (EGS) Patients and Their Proxies: A Single-Center Pilot Study. *Journal of the American College of Surgeons*, *237*(5), S169. https://doi.org/10.1097/xcs.0000000000000827 |
| 3 | Alben et al, 2023. Minimal clinically important diference, substantial clinical benefit, and patient acceptable symptom state of upper extremity PROMIS score sin idiopathic adhesive capsulities |
| 4 | Alpasan, M.C. (2024). The Relationship between Function, Stroke Severity and Perception of Health at Follow-up in Stroke Rehabilitation Patients. *Archives of Physical Medicine and Rehabilitation, 104*(4), e32. https://doi.org/10.1016/j.apmr.2024.02.087 |
| 5 | Anderson, AB, Tenan, MS, & Dickens, JG. Latent Factor Analysis of the PROMIS and Single Assessment Numeric Evaluation in Patients Undergoing Shoulder Surgery. *Military Medicine, 2021.* |
| 6 | Anwar et al, 2024. Impact of preoperative back pain severity on PROMIS outcomes following minimally invasive lumbar decompression. *European Spine Journal 33,* 4262-4269. https://doi.org/10.1007/s00586-024-08275-w |
| 7 | Arhewoh et al, 2025. Is There an Association Between Postoperative Internal Rotation and Patient-reported Outcomes After Total Shoulder Arthroplasty? *Clinical Orthopaedics and Related Research 483,* 152-159 https://doi.org10.1097/CORR.0000000000003199 |
| 8 | Armento A, Keeter C, Gagliardi A, et al. Association of Grit With Postoperative Knee Outcomes and Physical Function After ACL Reconstruction in Adolescent Athletes. The American Journal of Sports Medicine. 2023;51(11):2900-2907. doi:10.1177/03635465231187040 |
| 9 | Austin et al, 2018. Patient outcomes after total knee arthroplasty in patients older than 80 years. |
| 10 | Austin et al, 2019. A one-question patient-reported outcome measure is comparable to multiple-question measures in total knee arthroplasty patients. |
| 11 | Austin et al, 2019. Chronic condition measurement requires engagement, not measurement alone. |
| 12 | Azam, F., Shukla, I., Hicks, W. H., Anand, S., Furtado, K., Smith, P. D., Hall, K., Akbik, O. S., Bagley, C. A. (2024) A 3-Year Study Investigating the Utility of Patient-Reported Outcomes Measurement Information System-29 for Long-Term Follow-Up in Adult Spinal Deformity, World Neurosurgery, volume 186, Pages e506-e513, https://doi.org/10.1016/j.wneu.2024.03.165. |
| 13 | Babington et al, 2018. Patient-reported outcome measures: Utility for predicting spinal surgery in an integrated spine practice. |
| 14 | Bacalao, E. J., et al. (2017). Standardizing and personalizing the treat to target (T2T) approach for rheumatoid arthritis using the Patient-Reported Outcomes Measurement Information System (PROMIS): baseline findings on patient-centered treatment priorities. *Clinical Rheumatology (36),* 1729-1736. doi:10.1007/s10067-017-3731-5 |
| 15 | Barnett, N et al (2024) Testosterone and neurobehavioral outcomes in special operations forces military with multiple mild traumatic brain injury. *NeuroRehabilitation. 55*(3) 271-279 |
| 16 | Bartlett et al, 2015. Reliability and validity of selected PROMIS measures in people with rheumatoid arthritis. |
| 17 | Bartlett, S. J., et al. (2020). "Patient-reported outcomes in RA care improve patient communication, decision-making, satisfaction and confidence: qualitative results." Rheumatology (Oxford) 59(7): 1662-1670. |
| 18 | Bass et al, 2021. Implemention an application programming interface for PROMIS measures at three medical centers. (Center 1) |
| 19 | Bass et al, 2021. Implemention an application programming interface for PROMIS measures at three medical centers. (Center 2) |
| 20 | Bass et al, 2021. Implemention an application programming interface for PROMIS measures at three medical centers. (Center 3) |
| 21 | Bernstein et al, 2022. Sociodemographic factors are associated with patient-reported outcome measure completion in orthopaedic surgery |
| 22 | Bernstein, D. N., et al. (2020). "A Comparison of PROMIS Physical Function and Pain Interference Scores in Patients With Carpal Tunnel Syndrome: Research Collection Versus Routine Clinical Collection." Hand (N Y) 15(6): 771-775. |
| 23 | Bhandari, R. P., et al. (2016). Pediatric-Collaborative Health Outcomes Information Registry (Peds-CHOIR): A Learning Health System to Guide Pediatric Pain Research and Treatment. *Pain, 157*(9): 2033-2044, doi:10.1097/j.pain.0000000000000609 |
| 24 | Bhandari, R. P., et al. (2022). Clinical Utility of CAT Administered PROMIS Measures to Track Change for Pediatric Chronic Pain. The Journal of Pain |
| 25 | Bhatt, S, Davis, K, Manning, DW, Peabody, TD, Rothrock, NE. Integration of Patient-reported outcomes in a total join arthroplasty program at a high-volume academic medical center. |
| 26 | Biber et al, 2018. Patient reported outcomes - experiences with implementation in a University Health Care setting. Journal of Patient-Reported Outcomes. |
| 27 | Boden, A. L., Streeter, S. R., Masry, S. E., DiGiovanni, G., Cororaton, A., Conti, M. S., & Ellis, S. J. (2024). Defining the Patient Acceptable Symptom State (PASS) for PROMIS After Hallux Rigidus Correction Surgery. Foot & Ankle Orthopaedics, 9(2), 2473011424S00058. https://doi.org/10.1177/2473011424S00058 |
| 28 | Bouton 2022. Baseline patient reported outcome measurement information system (PROMIS) scores in children with idiopathic scoliosis and their relation to the SRS-22. |
| 29 | Bram, Joshua T. et al. Outcomes of grafting extracellular matrix with bone marrow aspirate concentrate in pediatric and adolescent patients with capitellar osteochondritis dissecans. Journal of Shoulder and Elbow Surgery, Volume 34, Issue 8, 2015 - 2021 |
| 30 | Burbank, A. J., Lucas, N., Lin, L., Hernandez, M. L., & Reeve, B. B. (2023). Measuring PROMIS Asthma Impact cut point scores for identifying uncontrolled asthma in children. The journal of allergy and clinical immunology. In practice, 11(12), 3783–3785. https://doi.org/10.1016/j.jaip.2023.08.045 |
| 31 | Call, C. M., Lachance, A. D., Zink, T. M., Stoddard, H., Babikian, G. M., Rana, A. J., & McGrory, B. J. (2025). Variation in Demographics, Hospital, and Patient-Reported Outcomes Following Total Hip Arthroplasty According to Biological Sex. The Journal of arthroplasty, 40(1), 127–135.e1. https://doi.org/10.1016/j.arth.2024.06.063 |
| 32 | Caolo et al, 2021. Clinical outcomes and complications with open vs minimally invasive achilles tendon repair. |
| 33 | Carpenter et al, 2022. An activity scale for all youth athletes? Clinical considerations for the HSS Pedi-FABS |
| 34 | Casson, et al. 2023. Implementation of Patient-Reported Outcomes Measurement Information System (PROMIS) to assess cognition in older elective hernia patients: A pilot study. Journal of the American College of Surgeons, 236(5), S50. |
| 35 | Cha et al, 2021. Impact of time to complete PROMIS-PF surveys on the scores of patients undergoing lumbar decompression. |
| 36 | Chang, H. R., Su, H. J., Kartawira, K., Grossberg, A., & Wan, J. (2025). Feasibility and Acceptability of Patient-Reported Outcomes Collection in Routine Pediatric Atopic Dermatitis Care. Pediatric dermatology, 10.1111/pde.15972. Advance online publication. https://doi.org/10.1111/pde.15972 |
| 37 | Chipman, D. et al Unique Activity Characteristics of Adolescents With Patellar Dislocations. Journal of ISAKOS, Volume 12, 100729 |
| 38 | Chrea et al, 2021. Clinical and patient-reported outcomes following peroneus brevis reconstruction with hamstring tendo autograft. |
| 39 | Como, C. J., Ramanathan, R., Dalton, J., Chang, A. Y., Gonzalez, C., Jr., Tang, M. Y., Adida, S., Calcaterra, M., Oyekan, A. A., Sadhwani, S., Chang, F., Ma, Y., Spitnale, M., Wawrose, R. A., Lee, J. Y., Shaw, J. D., & Fourman, M. S. (2025). Are hospital experience scores correlated with patient-reported outcome measures following elective spine surgery?. Journal of Neurosurgery: Spine, 43(2), 211-216. https://doi.org/10.3171/2025.2.SPINE241534 |
| 40 | Compton, Jocelyn et al., Outcomes of Acute Repair Versus Nonrepair of Zone I Flexor Digitorum Profundus Tendon Injuries. Journal of Hand Surgery, Volume 48, Issue 8, 832.e1 - 832.e6 |
| 41 | Conti et al., 2021. Preoperatieve Patient-reported outcome measures relationship with postoperative outcomes in flexible adult-acquired flatfoot deformity. |
| 42 | Conti et al., 2021. Preoperative PROMIS Physical Function Scores Predict Postoperative Outcomes Following Total Ankle Replacement |
| 43 | Cook, Kallen, Buckenmaier, at al. 2017. Evaluation of the validity and response burden of patient self-report measures of the pain assessment screening tool and outcomes registry (PASTOR). Military Medicine, 182 |
| 44 | Covarrubias, O., Andrade, N. S., Mo, K. C., Dhanjani, S., Olson, J., Musharbash, F. N., Sachdev, R., Kebaish, K. M., Skolasky, R. L., & Neuman, B. J. (2024). Abnormal Postoperative PROMIS Scores are Associated With Patient Satisfaction in Adult Spinal Deformity and Degenerative Spine Patients. Spine, 49(10), 689–693. https://doi.org/10.1097/BRS.0000000000004783 |
| 45 | Dalton, K., Lyall-Watson, S., Young, A., Bade, S., & Simons, M. (2025). Therapy-led model of care for simple, diagnostic-defined pediatric hand fractures can maximize service access and improve consumer outcomes: An implementation study of value-based healthcare using mixed-method design. Journal of hand therapy : official journal of the American Society of Hand Therapists, S0894-1130(25)00078-X. Advance online publication. https://doi.org/10.1016/j.jht.2025.05.006 |
| 46 | Danoff, S. K. et al., Remote Patient Monitoring of ILD Patients Using Oxygen. Am J Respir Crit Care Med 2024;209:A1793 |
| 47 | Devkumar, D., Zapata, K., Ma, Y., Jo, C. H., Ramo, B. (2025). PROMIS and ODI Tools: Clinically Useful Markers of Abnormal MRI Findings in Pediatric Patients with Back Pain. Journal of Bone and Joint Surgery 107A(18) 2015-2022. http://dx.doi.org/10.2106/JBJS.24.01404 |
| 48 | Dusek, J. A., et al. (2018). Rational for routine collection of patient reported outcomes during integrative medicine consultation visits. *Complementary Therapies in Medicine, (37)*, 43-49. doi.org/10.1016/j.ctim.2018.01.012 |
| 49 | Ernecoff, N. C., Weir, R., Rodriguez, A., Schulson, L. B., Edelen, M. O., & Hanmer, J. (2023). Electronic health record (EHR)-based PROMIS measures among neurology clinic decedents and survivors: a retrospective cohort analysis. Health and quality of life outcomes, 21(1), 95. https://doi.org/10.1186/s12955-023-02176-0 |
| 50 | Esaa, F., Prezzano J, Pentland A, Wolf JR. The utility of PROMIS domain measures in dermatologic care. 2021. |
| 51 | Evans et al 2020. Is social deprivation associated with PROMIS outcomes after upper extremity fractures in children? |
| 52 | Flens G, Terwee CB, Smits N, et al. Construct validity, responsiveness, and utility of change indicators of the Dutch-Flemish PROMIS Item Banks for Depression and Anxiety administered as computerized adaptive test (CAT): A comparison with the Brief Symptom Inventory (BSI) |
| 53 | Garcia, S. F., et al. (2019). "Implementing electronic health record-integrated screening of patient-reported symptoms and supportive care needs in a comprehensive cancer center." Cancer. |
| 54 | Gaufin, M., et al. (2019). "Practical Screening for Depression in Dermatology: Using Technology to Improve Care." Br J Dermatol. |
| 55 | Gedert, R., Ochocki, D., Kortam, N., Huang, S., Nagaraja, V., Chakrabarti, K., Ford, J., Garber, M., Lee, J., Ognenovski, V., Roofeh, D., Cella, D., & Khanna, D. (2025). A pilot trial of integrating the Patient-Reported Outcome Measurement Information System (PROMIS®) into rheumatology care. Clinical and experimental rheumatology, 43(1), 112–118. https://doi.org/10.55563/clinexprheumatol/fp914f |
| 56 | Generalova O, et al. Implementation of a cloud-based electronic patient-reported outcome (ePRO) platform in patients with advanced cancer. |
| 57 | George et al, 2021. Can PROMIS measures be used to create subgroups for patients seeking orthopaedic care? |
| 58 | Gerhardt, W. E., et al. (2018). "Systemwide Implementation of Patient-Reported Outcomes in Routine Clinical Care at a Children's Hospital." Joint Commission Journal on Quality and Patient Safety 44(8): 441-453. |
| 59 | Gibbons, L. E., et al. (2016). Suitability of the PROMIS alcohol use short form for screening in a HIV clinical care setting. *Drug and Alcohol Dependence, (164)*, 113-119. doi:10.1016/j.drugalcdep.2016.04.038 |
| 60 | Gold, H. T., et al. (2018). "Implementation and early adaptation of patient-reported outcome measures into an electronic health record: A technical report." Health Informatics J: 1460458218813710. |
| 61 | Gordon et al, 2021. Minimal clinically important difference, substantial clinical benefit, and patient acceptable symptom state of PROMIS upper extremity after total shoulder arthroplasty. |
| 62 | Gressel, G. M., et al. (2019). "Utilizing the Patient Reported Outcomes Measurement Information System (PROMIS(R)) to increase referral to ancillary support services for severely symptomatic patients with gynecologic cancer." Gynecol Oncol 152(3): 509-513. |
| 63 | Guattery, et al. (2018). Floor effect of PROMIS Depression CAT associated with ahsty completion in orthopaedic surgery patients. Clinical Orthopaedics and Related Research, 476(4), 696-703. |
| 64 | Hadley, S. M., Jr, Bergman, R., Peabody, J. J., Westvold, S. J., Filler, R., Mutawakkil, M., Patel, M., & Kadakia, A. R. (2025). Does syndesmotic fixation technique impact complication rates and functional outcomes measured by PROMIS scores following operative repair of ankle fractures?. Journal of orthopaedic surgery and research, 20(1), 730. https://doi.org/10.1186/s13018-025-06137-9 |
| 65 | Harrison, J.M., Ernecoff, N.C., Lai, JS. et al. Health system implementation of the PROMIS Cognitive Function Screener in the Medicare Annual Wellness Visit: framing as abilities versus concerns. J Patient Rep Outcomes 8, 43 (2024). https://doi.org/10.1186/s41687-024-00699-8 |
| 66 | Haskell, A. and T. Kim (2018). "Implementation of Patient-Reported Outcomes Measurement Information System Data Collection in a Private Orthopedic Surgery Practice." Foot Ankle Int 39(5): 517-521. |
| 67 | Hatchimonji et al., 2021. Beyond morbidity and mortality: The practicality of measuring patient-reported outcomes in trauma. |
| 68 | Haunschild et al 2020. Establishing the minimal clinically imporant difference, patient acceptable symptomatic state, and substantial clinical benefit of the PROMIS upper extremity questionnaire after rotator cuff repair. |
| 69 | Haupt ET, Porter GM, Blough C, Michalski MP, Pfeffer GB. Outcomes of Charcot-Marie-Tooth Disease Cavovarus Surgical Reconstruction. (2024). Foot & Ankle International. 45(11) 1175-1183. doi:10.1177/10711007241271283 |
| 70 | Haupt, E. T., Porter, G. M., Blough, C., Michalski, M. P., & Pfeffer, G. B. (2024). Outcomes of Charcot-Marie-Tooth Disease Cavovarus Surgical Reconstruction. Foot & ankle international, 45(11), 1175–1183. https://doi.org/10.1177/10711007241271283 |
| 71 | Hejazi, A., Willis, C., Ye, X., Youssef, J., Moebus, C., Goss, B., Cornwall, B., Brodke, D., McCormick, Z. L., Schaecher, K., & Brixner, D. (2024). The relationship of PROMIS physical function scores and healthcare resource utilization in patients treated for chronic low back pain. Interventional pain medicine, 3(4), 100522. https://doi.org/10.1016/j.inpm.2024.100522 |
| 72 | Heng et al, 2021. Can the Knee Outcome and Osteoarthritis Score (KOOS) function subscale be linked to the PROMIS Physical Function to crosswalk equivalent scores? |
| 73 | Herbert, L. and S. Hardy (2019). "Implementation of a Mental Health Screening Program in a Pediatric Tertiary Care Setting." Clin Pediatr (Phila) 58(10): 1078-1084. |
| 74 | Ho et al, 2016. Preoperative PROMIS scores predict postoperative success in foot and ankle patients. |
| 75 | Holbert et al, 2022. The impact of depression and anxiety on perioperative outcomes and patient-reported outcome measurement information system physical function after throracolumbar sugery |
| 76 | Holeman, T. A., Chester, C., Hales, J. B., Zhang, Y., Johnson, C. E., & Brooke, B. S. (2024). Long-term patient-reported outcomes among patients undergoing revascularization vs medical therapy for intermittent claudication. Journal of vascular surgery, 80(2), 466–477.e4. https://doi.org/10.1016/j.jvs.2024.03.455 |
| 77 | Holeman, T. A., Hales, J., Cizik, A. M., Zickmund, S., Kean, J., & Brooke, B. S. (2025). Factors that impact the implementation of patient reported outcomes in routine clinical care for peripheral artery disease from the patient perspective. Quality of life research : an international journal of quality of life aspects of treatment, care and rehabilitation, 34(3), 711–723. https://doi.org/10.1007/s11136-024-03842-6 |
| 78 | Horn, Reinke, Mather, et al. 2021. Electronic health record–integrated approach for collection of patient-reported outcome measures: a retrospective evaluation. PMCID: PMC8247208 PMID: 34193125 |
| 79 | Hu et al, 2020. Comparison of patient-reported outcomes for major pathologies of the forefoot, midfoot, hindfoot, and achilles using PROMIS |
| 80 | Hubbard et al., 2022. Establishing the patient acceptable symptom state in a nonshoulder hand and upper extremity population for the disabilities of the arm, shoulder, and hand and patient... |
| 81 | Hung et al, 2018. Establishing minimum clinically important difference values for the Patient-Reported Outcomes Measurement Information System Physical Function, hip disability and osteoarthritis... |
| 82 | Iturralde, E., et al. (2017). "Implementation of Depression Screening and Global Health Assessment in Pediatric Subspecialty Clinics." J Adolesc Health 61(5): 591-598. |
| 83 | Jacob et al, 2022. The effect of the severity of preoperative leg pain on patient-reported outcomes, minimum clinically important difference achievement, and patient satisfactiona fter minimally invasive transforaminal lumbar interbody fusion. |
| 84 | Jacobs et al 2020. The impact of patient-reported outcomes on loss to follow-up care after bariatric surgery. |
| 85 | Johnson M, Wukich DK, et al. The impact of hospitalization for diabetic foot infection on health-related quality of life: Utilizing PROMIS |
| 86 | Kasturi et al, 2018. Feasibility, validity, and reliability of the 10-item PROMIS short form in outpatients with systemic lupus erythemastosus. |
| 87 | Kasturi, S., Ahearn, E. L., Batterman, A., Horton, R., Kleinman, J., Rose-Smith, J., LeClair, A. M., & Mandl, L. A. (2023). Measuring What Matters: A Qualitative Study of the Relevance and Clinical Utility of PROMIS Surveys in Systemic Lupus Erythematosus. The Journal of rheumatology, jrheum.2023-0184. Advance online publication. https://doi.org/10.3899/jrheum.2023-0184 |
| 88 | Katzan IL, et al. Patient-reported outcomes predict future emergency department visits and hospital admissions in patients with stroke. *Journal of the American Heart Association.* |
| 89 | Katzan, I. L., et al. Added value of patient-reported outcome measures in stroke clinical practice. *Journal of the American Heart Association,* 2017;6. doi.org/10.1161/JAHA.116.005356 |
| 90 | Khanna, D., et al. (2012). Feasibility and construct validity of PROMIS and "Legacy" instruments in an academic scleroderma clinic. *Value in Health* 15, 128-134. doi.org/10.1016/j.jval.2011.08.006 |
| 91 | Khullar, O. V., et al. (2017). Pilot study to integrate patient reported outcomes after lung cancer operations into the society of thoracic surgeons database. Annals of Thoracic Surgery, 104, 245-253. doi.org/10.1016;j.athoracsur.2017.01.110 |
| 92 | Klapproth et al, 2022. Health state utility differed systematically in breast cancer patients between the EORTC QLU-C10D and the PROMIS preference score. |
| 93 | Klemt C, et al. Outcomes and risk factors associated with 2-stage reimplantation requiring an interim spacer exchange for periprosthetic joint infection. Journal of arthroplasty. 2021 |
| 94 | Koltsov et al, 2017. Validation of patient-reported outcome measurement information system computerized adaptive tests against the foot and ankle outcome score for 6 common foot and ankle pathologies. |
| 95 | Kostyun, Regina O. PhD; Witmer, Daniel K. MD; Lucchio, Peter PsyD; Solomito, Matthew J. PhD. The PROMIS-10 Global Mental Health T-Score: An Independent Predictor of In-Hospital Recovery Following a Total Hip Arthroplasty. Journal of the American Academy of Orthopaedic Surgeons ():10.5435/JAAOS-D-24-00430, December 3, 2024. \| DOI: 10.5435/JAAOS-D-24-00430 |
| 96 | Lapin & Katzan, 2022. PROMIS global health: potential utility as a screener to trigger construct-specific patient-reported outcome measures in clinical care. |
| 97 | Lapin et al, 2019. Association between patient experience with patient-reported outcome measurements and overall satisfaction with care in neurology. |
| 98 | Lapin et al, 2021. Patient-reported experience with patient-reported outcome measures in adult patients seen in rheumatology clinics. Quality of Life Research. |
| 99 | Lapin, B. R., et al. (2018). Accuracy of Linking VR-12 and PROMIS Global Health Scores in Clinical Practice. *Value in Health (21)*, doi.org/10.1016/j.val.2018.03.011 |
| 100 | Lapin, B., Li, Y., Englund, K., & Katzan, I. L. (2024). Health-Related Quality of Life for Patients with Post-Acute COVID-19 Syndrome: Identification of Symptom Clusters and Predictors of Long-Term Outcomes. Journal of general internal medicine, 39(8), 1301–1309. https://doi.org/10.1007/s11606-024-08688-9 |
| 101 | Lawrie et al., 2021. Is the Patient-Reported Outcome Measurement Information System feasible in bundled payment for care improvement total knee arthroplasty patients? |
| 102 | Leung, Y. W., et al. (2016). Feasibility and Diagnostic Accuracy of the Patient-Reported Outcomes Measurement information System (PROMIS) Item Banks for Routine Surveillance of Sleep and Fatigue Problems in Ambulatory Cancer Care. *Cancer, DOI: 10.1002/cncr.30134* |
| 103 | Li, H, Flynn, DM, et al. Pattern of cumulative treatment hours on pain impact and PROMIS outcomes. Military Medicine. |
| 104 | Li, J., et al. (2018). "Capturing a Patient-Reported Measure of Physical Function Through an Online Electronic Health Record Patient Portal in an Ambulatory Clinic: Implementation Study." JMIR Med Inform 6(2): e31. |
| 105 | Lizzio, V. A., et al. (2019). "Feasibility of PROMIS CAT Administration in the Ambulatory Sports Medicine Clinic With Respect to Cost and Patient Compliance: A Single-Surgeon Experience." Orthop J Sports Med 7(1): 2325967118821875. |
| 106 | MacGregor, K. R., Hartman, T. J., Nie, J. W., Zheng, E., Oyetayo, O. O., Massel, D. H., Sayari, A. J., & Singh, K. (2023). Poor patient-reported mental health correlates with inferior patient-reported outcome measures following cervical disc replacement. Acta neurochirurgica, 165(11), 3511–3519. https://doi.org/10.1007/s00701-023-05774-5 |
| 107 | Mercurio, A. M., Scott, E. J., Sugimoto, D., Christino, M. A., Coene, R. P., Gossman, E. C., Cook, D. L., Kocher, M. S., Kramer, D. E., Yen, Y. M., Micheli, L. J., & Milewski, M. D. (2024). Assessing the Impact of Psychological Readiness on Performance and Symmetry in Functional Testing After ACL Reconstruction in Pediatric and Adolescent Patients. Orthopaedic journal of sports medicine, 12(9), 23259671241274768. https://doi.org/10.1177/23259671241274768 |
| 108 | Merrill et al, 2018. Impact of depression on patient-reported outcome measures after lumbar spine decompression. |
| 109 | Michalik & Patel. 2021. Evaluation of transforaminal epidural steroid injections for discogenic anxial lumbosacral back pain utilizing PROMIS as an outcome measure. |
| 110 | Milewski, M. D., Traver, J. L., Coene, R. P., Williams, K., Sugimoto, D., Kramer, D. E., Kocher, M. S., Micheli, L. J., Yen, Y. M., & Christino, M. A. (2023). Effect of Age and Sex on Psychological Readiness and Patient-Reported Outcomes 6 Months After Primary ACL Reconstruction. Orthopaedic journal of sports medicine, 11(6), 23259671231166012. https://doi.org/10.1177/23259671231166012 |
| 111 | Moazzami et al, 2021. Validity and reliability of patient reported outcomes measurement information system computerized adaptive tests in systemic lupus erythematous. |
| 112 | Moura, L. M., et al. (2016). "Feasibility of the collection of patient-reported outcomes in an ambulatory neurology clinic." Neurology 87(23): 2435-2442. |
| 113 | Myhre, L., Olsen, Z., Li, H., Zhang, Y., Cizik, A. M., & Haller, J. (2023). Determining the clinical significance of the PROMIS physical function score in the setting of femur fractures. European journal of orthopaedic surgery & traumatology : orthopedie traumatologie, 33(6), 2277–2282. https://doi.org/10.1007/s00590-022-03417-6 |
| 114 | Neal, J., et al. Distress screening through Patient-Reported Outcomes Measurement Information System (PROMIS) at an academic cancer center and network site: Implementation of a hybrid model. *JCO Oncology Practice, 2021;17*(11), doi.org/10.1200/OP.20.00473 |
| 115 | Nelson et al. 2021. The clinical utility of a brief psychological stress measure (PROMIS) in youth with chronic pain. |
| 116 | Newmeyer, N., Zhong, L., Cheslock, M., Sison, S. D. M., Raman, V., Whyman, J. D., & Kim, D. H. (2023). Letter to the Editor: Utility of Serial Patient Reported Outcomes Measurement Information System (PROMIS®) Physical Function Assessment in a Geriatrics Outpatient Clinic. The Journal of frailty & aging, 12(4), 329–330. https://doi.org/10.14283/jfa.2023.32 |
| 117 | Nie et al, 2023. Impact of body mass index on PROMIS outcomes following lumbar decompression. |
| 118 | Nixon et al, 2017. PROMIS Pain Interference and physical function scores correlate with foot and ankle ability measure (FAAM) in patients with hallux vagus. |
| 119 | Nixon et al. 2020. Relationship of Press Ganey Satisfaction and PROMIS function and pain in foot and ankle patients. |
| 120 | Nyirenda, J., et al. (2019). "Feasibility Testing of Health Information Technology: Enabled Patient-Reported Outcome Measurement in the Home Health Setting." Home Healthc Now 37(4): 222-226. |
| 121 | Otegbeye et al, 2020. Preoperative PROMIS physical function and perioperative complication in major abdominal colorectal operations. |
| 122 | Padilla et al, 2019. Relationship between the patient-reported outcome measurement information system and traditional patient-reported outcomes for osteoarthritis. |
| 123 | Papuga, M. O., et al. (2018). "Large-scale clinical implementation of PROMIS computer adaptive testing with direct incorporation into the electronic medical record." Health Syst (Basingstoke) 7(1): 1-12. |
| 124 | Parrish JM, et al. The impact of comorbidity burden on postoperative PROMIS Physical Function following minimally invasive transforaminal lumbar interbody fusion. Clinical Spine Surgery 2020 |
| 125 | Patel et al, 2022. Impact of gender on postsurgical outcomes in patients undergoing anterior cervical discectomy and fusion. |
| 126 | Penedo et al. 2022. Implementation and feasibility of an electronic record-integrated patient-reported outcomes symptom and needs monitoring pilot in ambulatory oncology. DOI: 10.1200/OP.21.00706 |
| 127 | Philip et al 2021. Patient reported outcomes after risk-reducing surgery in patients at increased risk of ovarian cancer. |
| 128 | Porter RS, Holt K, & Ramchandran RS. Implementation of PROMIS in an optometry clinic. Patient Reported Outcome Measures. |
| 129 | Quinzi et al 2020; The impact of total hip arthroplasty surgical approach on patient-reported outcomes measurement information system computer adaptive tests of physical function and pain interference. |
| 130 | Rabah et al, 2020. The association between patient rating of their spine surgeon and quality of postoperative outcomes. |
| 131 | Riggs et al, 2021; PROMIS Global Health: Feasibility in home health. |
| 132 | Rizk, P. A., Silverberg, A., Deen, J., Pulido, L., Horodyski, M., Gray, C. (2023) Radiographic parameters of pelvic and proximal femoral morphology do not predict outcomes for direct anterior total hip arthroplasty, Injury, 54(10) 1-8 |
| 133 | Roca, A. M., Anwar, F. N., Khosla, I., Medakkar, S. S., Loya, A. C., Sayari, A. J., Lopez, G. D., & Singh, K. (2024). Utility of preoperative comorbidity burden on PROMIS outcomes after lumbar decompression: Cohort matched analysis. Journal of clinical neuroscience : official journal of the Neurosurgical Society of Australasia, 121, 23–27. https://doi.org/10.1016/j.jocn.2024.02.001 |
| 134 | Rocca, M. S., Honig, E. L., Tran, A., Kolevar, M. P., Kaveeshwar, S., Aneizi, A., Leong, N. L., Packer, J. D., Henn, R. F., 3rd, & Meredith, S. J. (2024). Preoperative opioid use is associated with worse preoperative patient-reported outcomes in hip arthroscopy patients. Journal of ISAKOS : joint disorders & orthopaedic sports medicine, 9(4), 581–586. https://doi.org/10.1016/j.jisako.2024.04.016 |
| 135 | Rotenstein et al., 2017. Implementing patient-reported outcome surveys as part of routine care: lessons from an academic radiation oncology department. |
| 136 | Rothrock, N. E., et al. (2019). "AO Patient Outcomes Center: Design, Implementation, and Evaluation of a Software Application for the Collection of Patient-Reported Outcome Measures in Orthopedic Outpatient Clinics." JMIR Form Res 3(2): e10880. |
| 137 | Rutherford et al., 2021. Implementing patient-reported outcome measures into clinical practice across NSW: Mixed methods evaluation of the first year. Applied Research in Quality of Life. |
| 138 | Saygin et al, 2021; Utility of patient-reported outcomes measurement information system (PROMIS) physical function form in inflammatory myopathy |
| 139 | Scholle, S. H., et al. (2018). "Implementation of the PROMIS-29 in Routine Care for People With Diabetes: Challenges and Opportunities." J Ambul Care Manage 41(4): 274-287. |
| 140 | Schwartzman et al, 2021; Validity and reliability of patient-reported outcomes measurement information system global health scale in adults with atopic dermatitis. |
| 141 | Seneviratne, M. G., et al. (2019). "Distribution of global health measures from routinely collected PROMIS surveys in patients with breast cancer or prostate cancer." Cancer 125(6): 943-951. |
| 142 | Shaffrey, I., Nguyen, J., Conti, M., Cody, E., Ellis, S., Demetracopoulos, C., & Henry, J. K. (2024). Defining the Patient Acceptable Symptom State (PASS) for PROMIS After Total Ankle Replacement. The Journal of bone and joint surgery. American volume, 106(15), 1404–1410. https://doi.org/10.2106/JBJS.23.01133 |
| 143 | Shaw et al, 2020. What does your PROMIS score mean? Improving the utility of patietn-reported outcomes at the point of care. |
| 144 | Shlykov et al, 2022. Does the Canadian orthopaedic foot and ankle society posoperative COFAS.... |
| 145 | Sidharthan et al, 2021; Bifocal patellar tendon avulsion fractures in children and adolescents: Diagnosis and treatment considerations for a unique injury pattern. |
| 146 | Sikorskii, A., Tam, S., Given, B., Given, C. W., Adjei Boakye, E., Zatirka, T., Nair, M., Su, W. K., Jogunoori, S., Watson, P., Movsas, B., & Chang, S. (2024). Thresholds in PROMIS Scores Anchored to Subsequent Unscheduled Health Service Use Among People Diagnosed With Cancer. JCO oncology practice, 20(10), 1391–1400. https://doi.org/10.1200/OP.23.00356 |
| 147 | Sisodia et al, 2020. Factors associated with increased collection of patient-reported outcomes within a large health care system. |
| 148 | Smit et al, 2021. A patient-reported outcomes measurement information system short form for measuring physical function during geriatric rehabilitation: Test-restest reliability, construct validity, responsiveness, and interpretability. |
| 149 | Solomito, M. J., Kostyun, R. O., & Makanji, H. (2024). The PROMIS-10 Mental Health T-Score: An Independent Predictor of Recovery for Up to 1 Year After Elective Lumbar Spine Fusion. Spine, 49(24), 1737–1742. https://doi.org/10.1097/BRS.0000000000005103 |
| 150 | Solomito, M. J., Makanji, H., & Eisler, J. (2025). Identifying patients at risk for a facility discharge following elective one or two level lumbar fusion. North American Spine Society journal, 24, 100786. https://doi.org/10.1016/j.xnsj.2025.100786 |
| 151 | Solomito, M. J., Witmer, D. K., Lucchio, P., & Kostyun, R. O. (2025). Utility of the Patient-Reported Outcomes Measurement Information System Global Mental Health T-Score to Identify Patients at Risk for Poor Postsurgical Outcomes Following Total Hip Arthroplasty. The Journal of arthroplasty, 40(4), 929–934. https://doi.org/10.1016/j.arth.2024.09.041 |
| 152 | Spraggs-Hughes, A., et al. (2018). "Clinical Application and Usage of Patient Reported Outcome Measures in an Orthopaedic Outpatient Setting." Value in Health (2018) 21: S357. |
| 153 | Stehlik, J., et al. (2017). "Implementation of Real-Time Assessment of Patient-Reported Outcomes in a Heart Failure Clinic: A Feasibility Study." J Card Fail 23(11): 813-816. |
| 154 | Streeter, S. R., Kush, S., Cororaton, A., Henry, J., Ellis, S. J., & Conti, M. S. (2024). Defining the Patient Acceptable Symptom State Using PROMIS Following Reconstruction of the Progressive Collapsing Foot Deformity. Foot & Ankle Orthopaedics, 9(2), 2473011424S00060. https://doi.org/10.1177/2473011424S00060 |
| 155 | Streller, Read BS*; Kelly, Brian A. MD*,†; Luhmann, Scott J. MD*,†. The Impact of Lumbar Partial Microdiscectomy for Vertebral Ring Apophyseal Fractures in Adolescents on PROMIS Pain, Physical Function, and Mental Health Domains. Journal of Pediatric Orthopaedics 45(8):p e711-e717, September 2025. \| DOI: 10.1097/BPO.0000000000002982 |
| 156 | Tamminga et al, 2020. Changes in the ability to participate in and satisfaction with social roles and activities in patients in outpatient rehabilitation. |
| 157 | Tucker, N. J., Shah, A., Mauffrey, C., Hammerberg, E. M., & Parry, J. A. (2023). Disconnected: Electronic Patient-Reported Outcome Measure Collection in Orthopaedic Patients is Less Successful Than In-Person Collection at an Urban Safety Net Trauma Center. Journal of orthopaedic trauma, 37(11S), S7–S11. https://doi.org/10.1097/BOT.0000000000002687 |
| 158 | Turcotte, J. J., Brennan, J. C., Baxter, S., Lashgari, A., Stock, L. A., Johnson, A. H., King, P. J., & Patton, C. M. (2023). Effect of Lower Extremity Osteoarthritis on Outcomes of Lumbar Decompression. Journal of the American Academy of Orthopaedic Surgeons. Global research & reviews, 7(10), e23.00044. https://doi.org/10.5435/JAAOSGlobal-D-23-00044 |
| 159 | Turcotte, J. J., Brennan, J. C., Johnson, A. H., Patton, C. M., & Gelfand, J. (2025). Examining the Relationship Between Relative Value Units and Patient-reported Outcomes: Implications for Value-based Care. The Journal of the American Academy of Orthopaedic Surgeons, 33(11), 602–609. https://doi.org/10.5435/JAAOS-D-24-01297 |
| 160 | Urbonas, R., et al (2025). Journal of Investigative Dermatology. Volume 145, Issue 8, S45 |
| 161 | Wagner, L. I., et al. (2015). "Bringing PROMIS to practice: brief and precise symptom screening in ambulatory cancer care." Cancer 121(6): 927-934. |
| 162 | Wahl E, Gross A, Chernitsky V, Trupi, L, Gensler LS, Chaganti K, Michaud K, Katz P, & Yazdany, J. Validity and responsiveness of a 10-item patient-reported measure of physical function, PROMIS PF-10a, in a rheumatoid arthritis clinic population. *Arthritis Care Res.* 2017;69(3):338-346. doi:10.1002/acr.22956 |
| 163 | Webster, C. E., Stiles, E., Scotti, A., Kim, Y. H., & Fischer, C. R. (2025). Health Literacy and Patient-Reported Outcomes Measurement Information System Scores Among Patients Referred to Spine Surgeons. Cureus, 17(3), e80717. https://doi.org/10.7759/cureus.80717 |
| 164 | Zeng C, Martin NE, Pusic AL, et al. Enhancing representativeness of patient-reported outcomes in routine radiation oncology care: a quality improvement protocol to address non-response. BMJ Open 2024;14:e097127. doi:10.1136/bmjopen-2024-097127 |
| 165 | Zeng, C., Kaur, M. N., Malapati, S. H., Liu, J. B., Bryant, A. S., Meyers, P. M., Bates, D. W., McCleary, N. J., Pusic, A. L., & Edelen, M. O. (2024). Patterns of Social Needs Predict Quality-of-Life and Healthcare Utilization Outcomes in Patients from a Large Hospital System. Journal of general internal medicine, 39(11), 2060–2068. https://doi.org/10.1007/s11606-024-08788-6 |
| 166 | Ziedas et al, 2023. Associating social determinants of health with PROMIS CAT scores and health care utilization after ACL reconstruction. |
| 167 | Ziegenfuss et al. 2022. Is the Promise of PROMs being realized? Implementation experience in a large orthopedic practice. |

**Conference Abstracts**

| # | Citation |
| --- | --- |
| 1 | Abarca, Johnson, Tolbert, & James. (2018) The implementation of PROMIS at Shriners Hospitals for Children Northern California: A patient reported outcomes study. PROMIS Health Organization Conference. |
| 2 | Abarca, Johnson, Tolbert, Aubert, & James. (2019) The implementation of patient repoted outcomes in multi-disciplinary outpatient clinics. PROMIS Health Organization Conference. |
| 3 | Ahmed, Edwards, Amiri, ... Mucsi. (2022) Two-step screning for depressive symptoms in solid organ transplant recipients. JPRO, 7(1): O82. PROMIS Health Organization Conference. |
| 4 | Anderson, Baumhauer, et al. (2017) Clinical utilization of patient reported outcomes (PROMIS) scores for surgical reconstruction of postereior tibialis tendon dysfunction. 1. PROMIS Health Organization Conference. |
| 5 | Anderson, Baumhauer, et al. (2017) The ability of PROMIS scales to predict Patient Acceptable Symptom State (PASS) at an average of 14 month follow up after foot and ankle surgery. 2. PROMIS Health Organization Conference. |
| 6 | Antalis, Reinke, et al. (2019) Using PROMIS tools to assess change in patients' experiences of anxiety, depression, and pain among cancer survivors in a survivorship clinic. P26. Health Measures User Conference. |
| 7 | Antao, Fontana, So, Maclean. (2021) Assocations between workers' compesnation status and preoperatie PROMIS Global Health scores in orthopedic patients. JPRO 5(1): O15. PROMIS Health Organization Conference. |
| 8 | Antao, Ghanta, Chicosky, Gantha, MacLean.(2022) Challenges and successes in the implementation of PROMs at a musculoskeletal specialty hospital. Journal of Patient-Reported Outcomes 2023, 7(1):O20. PROMIS Health Organization Conference. |
| 9 | Bailes, A. H., Johnson, M. E., Redfern, M., Perera, S., Greco, C., Sowa, G., Cham, R., & Bell, K. (2023). 283 Gait: a biomarker for pain and function in chronic low back pain. Journal of Clinical and Translational Science, 7(Suppl 1), 85. https://doi.org/10.1017/cts.2023.339 |
| 10 | Bailey et al (2024) Implementation of paient-reported outcome measures in psychiatric urgent care. PROMIS Health Organization Conference. |
| 11 | Bao, Keeney, et al. (2017) Evaluating the impact of orthopaedic resident participation and postgraduate year on patient-reported outcomes using PROMIS-10. 3. PROMIS Health Organization Conference. |
| 12 | Bartlett S et al (2024) PROMIS symptom clusters at diagnosis predict poor treatment response at 6 months in rheumatoid arthritis. PROMIS Health Organization Conference. |
| 13 | Bartlett, DiRenzo, Jones, & Bingham. (2021) Many better, many worse: mean PROMIS-29 Scores mask significant shifts during COVID-19 in RA. JPRO 5(1): O75. PROMIS Health Organization Conference. |
| 14 | Bartlett, Jones, et al. (2017) Minimally clinically important differences of PROMIS measures vary by symptom, direction, and level of disease activity in theumatoid arthritis. 105.4. ISOQOL. |
| 15 | Bartlett, Orbai, et al. (2013) Validity of PROMIS CATs, short forms and global health items in rheumatoid arthritis. 3024. ISOQOL. |
| 16 | Bartlett, Orbai, et al. (2015) Integrating PROs into routine arthritis clinical care: process and feasibility. Symposium 2. ISOQOL. |
| 17 | Bartlett, Orbal, et al. (2015) How well do generic patient PROMIS instruments capture health status in rheumatoid arthritis? PROMIS Health Organization Conference. |
| 18 | Bartosiak, Bernholt, et al. (2018) Inter-provider and problem-specific variability of PROMIS-CAT patient reported scores. P004. PROMIS Health Organization Conference. |
| 19 | Baumhauer, Anderson, et al. (2017) Generalizability and validation of PROMIS scores to predict surgical success in foot and ankle patients. P2. Health Measures User Conference. |
| 20 | Baumhauer, Anderson, et al. (2017) Generalizability and validation of PROMIS scores to predict surgical success in foot and ankle patients: A tale of two academic enters. 7. PROMIS Health Organization Conference. |
| 21 | Baumhauer, Flemister, et al. (2018) Can women live with more symptoms than men? Defining the Patient Acceptable Symptom State (PASS). P005. PROMIS Health Organization Conference. |
| 22 | Baumhauer, Ho, et al. (2016) Impact of depression on physical function and pain following foot and ankle fracture. 41. PROMIS Health Organization Conference. |
| 23 | Baumhauer, Kohring, et al. (2019) How well do patients recover compared to population norms after an ankle arthrodesis surgery? P6. PROMIS Health Organization Conference. |
| 24 | Baumhauer, Teitel, et al. (2018) Identifying foot and ankle patients at risk to fall based on patient reported outcome assessments. S10. ISOQOL. |
| 25 | Beleckas, Gerull, et al. (2017) Utilization after carpal tunnel release PROMIS predicts increased resource. 10. PROMIS Health Organization Conference. |
| 26 | Beleckas, Gerull, et al. (2018) Predicting increased resource utilization after carpal tunnel release. P006. PROMIS Health Organization Conference. |
| 27 | Beleckas, Guattery, et al. (2017) PROMIS captures variability in physical function across hand conditions. 9. PROMIS Health Organization Conference. |
| 28 | Beleckas, Prather, et al. (2018) Anxiety in the orthopaedic patient: Using PROMIS to assess mental health. P007. PROMIS Health Organization Conference. |
| 29 | Bernstein, Lans, ... Tobert. (2022) The association of granular, patient-level social determinant of health factors on presenting PROMIS Global-10 scores. JPRO, 7(1): O63. PROMIS Health Organization Conference. |
| 30 | Bernstein, Mayo, et al. (2019) Using PROMIS to determin if the patient acceptable symptom state differs by socioeconomic status. JPRO, 3(S1):O4. PROMIS Health Organization Conference. |
| 31 | Bhatt, Kutay, et al. (2017) Incorporating PROs into the EMR: The Northwestern Medicine orthopaedic surgery joint replacement pilot. P4. Health Measures User Conference. |
| 32 | Biber, Hess, et al. (2016) Implementing patient reported outcomes throughout a healthcare system. 3059. ISOQOL. |
| 33 | Biber, Hess, et al. (2017) Comparing automated mental health screening to manual processes in a health care system. 201.4. ISOQOL. |
| 34 | Biber, Hess, et al. (2017) Comparing mental health to physical functioning. 2062. ISOQOL. |
| 35 | Biber, Hess, et al. (2017) Reviewing mental health in oncology. 201.3. ISOQOL. |
| 36 | Biber, Weeks, et al. (2018) Change in PROMIS physical function scores for bariatric surgery. 2109. ISOQOL. |
| 37 | Biber, Weeks, et al. (2018) Mapping PROMIS physical function scores to functional ability. 107.1. ISOQOL. |
| 38 | Bingham & Bartlett. (2013) Integrating patient-centered outcomes in arthritis clinical care. Symposium 1. ISOQOL. |
| 39 | Biswas, A., Zamore, Z. H., Aslami, Z. V., Tiongco, R. F. P., Ali, A., Cooney, C. M. M., Fisher, M., Caffrey, J. A., & Lerman, S. F. (2024). 51 The Association Between Neighborhood Disadvantage and Patient-Reported Outcomes in Burn Survivors. Journal of Burn Care & Research: Official Publication of the American Burn Association, 45(Suppl 1), 35. https://doi.org/10.1093/jbcr/irae036.075 |
| 40 | Black, Castro, & Vrahas. (2019) The EPIC PROMIS: implementing routine PROMS collection in orthopaedics. P11 PROMIS Health Organization Conference. |
| 41 | Brinker et al. (2024) Linking algorithms based on the PROMIS Wave 1 sample can be used to translate SF-36 PF-10 scores to the PROMIS Physical Function metric in independent clinical samples. ISOQOL. |
| 42 | Burckel, Baumhauer, et al. (2018) PROMIS Physical Function scores predict discharge to home following transcatheter and surgical aortic valve replacement. O011. PROMIS Health Organization Conference. |
| 43 | Bydon 2020. Feasibility of using Computerized Adaptive Testing to Capture Patient Reported Outcomes in an Outpatient Setting: A Pilot Evaluation of PROMIS-CAT in Neurosurgery. J Neurosurg v 132. |
| 44 | Calfee, Guattery, et al. (2016) Patient-reported musculoskeletal function: Comparing PROMIS upper extremity to PROMIS general physical function scores. 68. PROMIS Health Organization Conference. |
| 45 | Caplin et al (2023) Sleep well, stay well? Multi-informant assessment of pediatric sleep-related impairment and behavioral health referrals. PROMIS Health Organization Conference. |
| 46 | Castle J et al (2023) Race, sex, and income negatively impact patient-reported outcomes following total shoulder arthroplasty. PROMIS Health Organization Conference. |
| 47 | Castle J et al (2023) Surprising health state satisfaction prior to total hip and knee arthroplasty. PROMIS Health Organization Conference. |
| 48 | Castro & Vrahas. (2020) Effectiveness of automated EMR notification for prompting provider intervention for severe depression. JPRO 4(S2):167-O. PROMIS Health Organization Conference. |
| 49 | Chamberlain, Calfee, et al. (2016) Correlation of PROMIS upper extremity, pain interference, and depression scores with SST and ASES scores. 44. PROMIS Health Organization Conference. |
| 50 | Cizik, Sangeorzan, & Kleweno. (2017) Measuring patient-reported outcomes (PROs) in a high volume trauma clinic in pelvis and acetabular facture patients using PROMIS CAT: A preliminary study. 40. PROMIS Health Organization Conference. |
| 51 | Collins. (2019) Evaluating the social health of pediatric patients using PROMIS Peer Relationships: Social work intervention thresholds. P48. Health Measures User Conference. |
| 52 | Cook, Kallen, et al. (2015) Uncle Sam wants PROMIS: Leveraging PROMIS to build and pilot the DoD's Pain Assessment Screening Tool and Registry (PASTOR). PROMIS Health Organization Conference. |
| 53 | Cottom, Guarino, & Sellers. (2019) Integrating pediatric PROMIS measures into clinical and technology operations of Shriners Hospitals for Children health system. p38. Health Measures User Conference. |
| 54 | Cox et al. (2024) Postoperative quality of life in thoracic surgery patients using electronic patient-reports outcomes. ISOQOL. |
| 55 | Crangle, Dannenberg, & Hamel. (2019) Using PROMIS in batterer intervention programs: Towards program evaluation. P10. Health Measures User Conference. |
| 56 | Dano, Tang, et al. (2019) Measurement characteristics of PROMIS Computer Adaptive Testing (CAT) fatigue and ESASr tiredness in kidney transplant. O16. PROMIS Health Organization Conference. |
| 57 | Dardas, Chamberlain, et al. (2016) Variability of PROMIS domains across orthopedic conditions. 67. PROMIS Health Organization Conference. |
| 58 | Dardas, Guattery, et al. (2016) Floor effect of depression on patients with orthopedic conditions. 66. PROMIS Health Organization Conference. |
| 59 | DiRenzo, Robinson, Bingham, Baer, Grader-Beck. (2021) PROMIS provides a broader overview of HRQL than the ESSPRI in Sjogren's Syndrome. JPRO 5(1): P21. PROMIS Health Organization Conference. |
| 60 | DiRenzo, Wu, et al. (2019) Anxiety and depression symptoms increase office telephone communication for patients with rheumatoid arthritis. P9. PROMIS Health Organization Conference. |
| 61 | Donohoe, McGreal, ... Wellmon. (2020). Measuring function in a multidisciplinary Osteogenesis Imperfecta clinic. JPRO 4(S2):114-P. PROMIS Health Organization Conference. |
| 62 | Doorly & Sisodia. (2019) Preoperative PROMIS 10 physical function scores help predict opioid dependence after lumbar fusion surgery. O18. PROMIS Health Organization Conference. |
| 63 | Edelen M. et al (2023) PROMIS Cognitive Function abilities versus concerns in the Medicare Annual Wellness Visit. PROMIS Health Organization Conference. |
| 64 | Edelen, Hanmner, ... Dana. (2021) Association of PROMIS-Cognitive Function Scores with the Montreal Cognitive ASsessment Test in a clinical sample. JPRO 5(1): P64. PROMIS Health Organization Conference. |
| 65 | Fadlallah, J et al (2024) Physical fucntion recovery following kidney transplantation. PROMIS Health Organization Conference. |
| 66 | Fear, Dasilva, et al. (2019) Evaluating clinical impact of a standard error reduction (SER) based on stopping rule for PROMIS CAT instruments. P35. Health Measures User Conference. |
| 67 | Fear, Teitel, et al. (2018) Identifying foot and ankle patients at risk to fall based on patient reported outcome assessments. O024. PROMIS Health Organization Conference. |
| 68 | Fisk, Franovic, et al. (2019) PROMIS forms demonstrate responsiveness in patients following arthroscopic rotator cuff repair across numerous health comains. P36. PROMIS Health Organization Conference. |
| 69 | Flens, Roorda, et al. (2016) Feasibility and reliability of the Dutch-Flemish PROMIS Computer Adaptive Tests for depression and anxiety. 2. PROMIS Health Organization Conference. |
| 70 | Fontana, Antao, So, & Maclean. (2021) Utility of interpretable general additive models in predicting PROMIS Global Health scores in orthopedic patients. JPRO 5(1): P42. PROMIS Health Organization Conference. |
| 71 | Forrest. (2014) Integrating computerized adaptive tests into electronic health records and clinical care. Symposium 3. ISOQOL. |
| 72 | Frank J. Penedo et al. Ethnic disparities in unmet supportive care needs and outcomes among older ambulatory cancer patients.. J Clin Oncol 43, 11108-11108(2025). DOI:10.1200/JCO.2025.43.16_suppl.11108 |
| 73 | Gagnier, Lee, et al. (2017) PROMIS Global Health scale in patients undergoing total joint arthroplasty. 17. PROMIS Health Organization Conference. |
| 74 | Garcia, Penedo, et al. (2017) Implementing PROMIS for routine screening in ambulatory cancer care. P15. Health Measures User Conference. |
| 75 | Gaskins et al (2023) Examining recovery trajectories of patients with burn injuries using the PROMIS10 global health survey. PROMIS Health Organization Conference. |
| 76 | Ghazi, Wen, Alhabbal, ... Mucsi. (2022) Association of PROMIS physical function and health-related quality of life among solid organ transplant recipients. JPRO, 7(1): P81. PROMIS Health Organization Conference. |
| 77 | Gill et al (2023) The association between fatigue and physical functioning in patients with kidney failure. PROMIS Health Organization Conference. |
| 78 | Gill et al. (2023) Moderate/severe fatigue is associated with impaired physical function among patients in kidney failure. ISOQOL. |
| 79 | Gold, Link, et al. (2017) Technical evaluation of impelmenting patient-reported outcomes measures into an electronic health record. P15. Health Measures User Conference. |
| 80 | Griffith & Katzan. (2014) An interactive web-based application for displaying population-specific comparisons using PROMIS scores. 202.5. ISOQOL. |
| 81 | Groe K, et al (2024) Trajectory of anxiety following liver transplantation. PROMIS Health Organization Conference. |
| 82 | Gruner, Nyman, Wolff, Sellon, Gonzalez. (2020) Feasibility using PROMIS-CAT in a sports medicine center and regenerative medicine registry in outpatient setting. JPRO 4(S2):121-O. PROMIS Health Organization Conference. |
| 83 | Guattery & Calfee. (2016) Improving and expediting access to PROMIS CAT data in orthopaedic practice. 26. PROMIS Health Organization Conference. |
| 84 | Guattery, Spraggs-Hughes, et al. (2018) Implementing and integrating patient reported outcomes data capture in an academic medical center setting. P028. PROMIS Health Organization Conference. |
| 85 | Guattery, Spraggs-Hughes, et al. (2018) Lessons learned and future directions for patient reported outcome usage at an academic medical center. P029. PROMIS Health Organization Conference. |
| 86 | Gulledge, Korn, et al. (2019) Examining predictors of achieving MCID following ACL reconstruction: the importance of preoperative PROMIS CAT scores. P72. PROMIS Health Organization Conference. |
| 87 | Gulledge, Smith, et al. (2019) Floor and ceiling effects of PROMIS in sports medicine patients undergoing non-operative and operative treatment. P37. PROMIS Health Organization Conference. |
| 88 | Guo, Cross, ... Makhni. (2020) Presence of preoperative clinical depression does not hinder recovery after anterior cruciate ligament reconstruction. JPRO, 4(S2):139-P. PROMIS Health Organization Conference. |
| 89 | Guo, Elhage, ... Makhni. (2020) Establishing and comparing reference pre-operative PROMIS score in patients undergoing shoulder surgery. JPRO, 4(S2):140-P. PROMIS Health Organization Conference. |
| 90 | Haggstrom, Talib, et al. (2017) Incorporating PROMIS symtpom measures into primary care practice: A randomized trial. P18. Health Measures User Conference. |
| 91 | Hanmer, Dunn, et al. (2019) PROMIS domain scores are associated with utilization in an outpatient sample of neurology patients. P27. Health Measures User Conference. |
| 92 | Hanmer, Freel, & Liposky. (2019) UPMC patient reported outcomes center's governance structure, integration with Epic, and clinical operations training. P4. Health Measures User Conference. |
| 93 | Hartmann & Fischer. (2019) Reducing respondent burden for spine patients - developing a prediction model for ODI and COMI based on PROMIS 29. P20. PROMIS Health Organization Conference. |
| 94 | Hartmann, Fischer, et al. (2021) PROMIS-29 and EORTC QLQ-C30- an empirical investigation towards a common conception of health. 3038. ISOQOL. |
| 95 | Heinle IV, Suk, et al. (2019) Clinical utilization of patient reported outcomes in the upper extremity orthopaedic population: comparing the PROMIS upper extremity bank v2.0 and the QuickDASH measures. P23. PROMIS Health Organization Conference. |
| 96 | Hennekes, M. et al (2023) Timing of preoperative patient-reported outcome measures matters in patients undergoing anterior cruciate ligament reconstruction. PROMIS Health Organization Conference. |
| 97 | Hennekes, M. et al (2023) What does depression screening in the orthopedic clinic teach us? Results from nearly 60,000 patients. PROMIS Health Organization Conference. |
| 98 | Hesketch, Bannister, et al. (2015) Collecting outcomes in a high-throughput outpatient clinic: Significant and practicality of PROMIS CAT implementation. PROMIS Health Organization Conference. |
| 99 | Hess. (2020) Governing PROs across health systems: case study at University of Utah. S14. ISOQOL. |
| 100 | Ho. (2016) Initial PROMIS scores predict outcome in foot and ankle patients. Symposium 6. ISOQOL. |
| 101 | Holeman T et. al (2023) Implementation of patient-reported outcomes in routine clinical care: The patients' perspective. PROMIS Health Organization Conference. |
| 102 | Holleran, Baumhauer, ... Ketz. (2021) Is there a difference in outcomes between double or triple arthrodesis for foot deformity? JPRO 4(S2):104-P. PROMIS Health Organization Conference. |
| 103 | Holleran, Flemister, ... Baumhauer.(2020) Is there a difference in outcomes between patients treated with different implants for hammertoe correction? JPRO 4(S2):103-P. PROMIS Health Organization Conference. |
| 104 | Hopkins, Z., Stone, C., Broadbent, E., Sahni, V., Grey, L., Kean, J., Secrest, A., Hull, C., Rhoads, J., Hansen, C., Clarke, J., et al. Mental Health Diagnosis Impacts Measures of Resilience and Health-Reported Quality of Life, Journal of the American Academy of Dermatology, Volume 91, Issue 3, AB256 |
| 105 | Horn M et al (2024) Using PROMIS measures to estimate high impact chronic pain (HICP) following total joint arthroplasty. PROMIS Health Organization Conference. |
| 106 | Houck, Ho, et al. (2016) Comparison of PROMIS physical function, pain interference, and depression at initial clinical assessment of foot and ankle soft tissue injuries. 43. PROMIS Health Organization Conference. |
| 107 | Houck, Ho, et al. (2016) PROMIS physical function, pain interference, and depression of total hip replacement participants pre-surgically to 6 months post operation. Symposium 6. ISOQOL. |
| 108 | Houck, Ho, et al. (2016)Comparison of patient reported outcomes 6-13 months after ankle fractuce and hip fracture. 42. PROMIS Health Organization Conference. |
| 109 | Houck, Konstantinos, et al. (2018) Does the Patient Reported Outcomes Information System (PROMIS) physical function scale perform as well as recommended performance based tests to identify older adults at falls risk? S9. ISOQOL. |
| 110 | Houck, Mayo, et al. (2018) Influence of socioeconomic factors and Patient Reported Outcome Measurement Information System on Patient Acceptable Symptom State (PASS). P033. PROMIS Health Organization Conference. |
| 111 | Houwen, Lansink, & Jongh. (2022) From numbers to meaningful change: minimal important change by using PRPOMIS in fracture patients. JPRO, 7(1): P41. PROMIS Health Organization Conference. |
| 112 | Hung, Baumhauer, et al. (2018) Identification of MCIDs for foot and ankle patients. 3049. ISOQOL. |
| 113 | Hung, Baumhauer, et al. (2018) Item response theory linking of the PROMIS and FAAM instruments. 3048. ISOQOL. |
| 114 | Hung, Greene, et al. (2017) Psychometrics and crosswalks of the PROMIS measures. 2041. ISOQOL. |
| 115 | Hung, Hon, et al. (2018) Crosswalks of the PROMIS PF and legacy sclaes across orthopaedic sub-specialties. O036. PROMIS Health Organization Conference. |
| 116 | Hung, Saltzman, et al. (2017) Utility of the PROMIS instruments in orthopaedics. 2047. ISOQOL. |
| 117 | Hung, Voss, et al. (2017) Evaluating change in joint function: The HOOS JR, the KOOS JR, and the PROMIS PF. P21. Health Measures User Conference. |
| 118 | Hung, Voss, et al. (2018) MCID values of the PROMIS PF and PI in orthopaedics. P035. PROMIS Health Organization Conference. |
| 119 | Hung. (2015) Challenges and lessons learned in developing a patient-reported outcomes clinical research program. PROMIS Health Organization Conference. |
| 120 | Hunt, Heatly, & Alpert-Gillis. (2021) Implementation of PROMIS CAT Pediatric Measures in outpatient behavioral health: adaptations and challenges during a pandemic. JPRO 5(1): P56. PROMIS Health Organization Conference. |
| 121 | Hunter, M., Gaskins, D., Hubbard, M., Carrougher, G., Wiechman, S., Stewart, B., Chambers, M., & Gibran, N. (2023). 731 Evaluation of PROMIS-10 Global Health Survey Implementation at a Regional Burn Center Clinic. Journal of Burn Care & Research: Official Publication of the American Burn Association, 44(Suppl 2), S144–S145. https://doi.org/10.1093/jbcr/irad045.206 |
| 122 | Hunter, Ramirez, Thirukumaran, Baumhauer. (2022) Can PROMIS scores be used to eliminate unnecessary follow up clinic visits after surgery? JPRO, 7(1): O25. PROMIS Health Organization Conference. |
| 123 | Hynes K et al (2024) Electronic Health Literacy (eHEALS) - a barrier to PROMIS CAT questionnaire completion in orthopaedic surgery. PROMIS Health Organization Conference. |
| 124 | Iyer, McGuire, Valencia, Beckwith, & Jo. (2021) Impact of COVID-19/social distancing on PROMIS pediatric peer realtionship T-scores in children with Legg-Calve-Perthes Disease. JPRO 5(1): P34. PROMIS Health Organization Conference. |
| 125 | Jacobson, Long, Kang, Amendola, & Houck. (2021) PROMIS Physical Function severity is associated with physical therapy recommendations in primary care. JPRO 5(1): O53. PROMIS Health Organization Conference. |
| 126 | Jevotovsky, Thirukumaran, & Rubery. (2018) The impact of lumbar discectomy on patient-reported outcomes: A matched cohort study. O039. PROMIS Health Organization Conference. |
| 127 | Joeris, Vrahas, & Rothrock. (2016) AO Patient Outcomes Center - A novel software to implemented PROMIS in orthopedic surgeons' clinical routine. 10. PROMIS Health Organization Conference. |
| 128 | Kaczmarek, Sethi, ... Gudesblatt. (2021) Relationship of physical ability in Multiple Sclerosis to patient reported meaning & purpose. JPRO 5(1): P45. PROMIS Health Organization Conference. |
| 129 | Kang, Cuddeford, Rahkola, & Houck. (2019) What are provider/patient PROMIS Scales as an assessment in primary care? P29. PROMIS Health Organization Conference. |
| 130 | Karia, Gold, et al. (2017) Feasibility and implemenation of PROMIS CAT as part of standard of care in an orthopaedic spine population. 75. PROMIS Health Organization Conference. |
| 131 | Karia, Gold, et al. (2017) Feasibility and implementation of PROMIS CAT as part of standard of care in an orthopaedic spin population. P23. Health Measures User Conference. |
| 132 | Kasturi, Koltsov, et al. (2015) Feasibility and validity of PROMIS in systemic lupus erythematosus (SLE). PROMIS Health Organization Conference. |
| 133 | Kasturi, Price, Curtis, ... McAlindon. (2021) Implementation of PROMIS Computerized Adaptive Tests in Systemic Lupus Erythematosus (SLE) clinical care. JPRO 5(1): O61. PROMIS Health Organization Conference. |
| 134 | Katzan, Lapin, Thompson, Russman, & Uchino .(2021) Implementation of PROMIS scales in a cerebrovascular clinic - experience after 10 years. JPRO 5(1): O671. PROMIS Health Organization Conference. |
| 135 | Katzan, Schuster, et al. (2018) Patient reported outcomes across stroke subtypes and TIA - is there a difference? 1012. ISOQOL. |
| 136 | Katzan, Thompson, et al. (2021) PROs to predict hospital admissions and ED visits. 103.3. ISOQOL. |
| 137 | Katzan, Youran, et al. (2015) Use of scale linkage to determine performance of PRO-PMs assessed with different patient-reported outcome measures. 302.1. ISOQOL. |
| 138 | Kaur et al (2023) Do social determinants ofhealth-related needs account for ethnoracial inequities in patient-reported mental health? PROMIS Health Organization Conference. |
| 139 | Kaur et al. (2023) How much do social determinants of health-related needs account for ethnoracial inequities in patient-reported mental health? ISOQOL. |
| 140 | Kerlin. (2019) Using PROMIS and NEuro-QoL in residential drug treatment facilities for both client assessment and program evaluation. P18. Health Measures User Conference. |
| 141 | Kohring, Humphrey, et al. (2019) Early improvement in physical function after symptomatic syndesmotic screw removal. P5. PROMIS Health Organization Conference. |
| 142 | Lai et al (2023) Exploring factors associated with trajectories of PROMIS Cognition Function over time. PROMIS Health Organization Conference. |
| 143 | Lai et al. (2024) A symptom monitoring and reporting program to improve health-related quality of life for children with cancer. ISOQOL. |
| 144 | Lapin & Katzan. (2021) PROMIS Global Health: potential utility as a screener to trigger construct-specific PROs in clinical care. JPRO 5(1): O40. PROMIS Health Organization Conference. |
| 145 | Lapin & Katzan. (2021) The imapct of COVID-19 on health-related quality of life: a pre-post cohort study with a propensity score matched control group. 104.1. ISOQOL. |
| 146 | Lapin B et al (2024) Measuring PROMIS global health across neurological populations. PROMIS Health Organization Conference. |
| 147 | Lapin et al. (2024) Neighborhood socioeconomic disadvantage and geographic disparities in global health. ISOQOL. |
| 148 | Lapin, Honomichi, et al. (2020) Patient-reported experience with patient-reported outcomes in adult patients seen in rheumatology clinics. 106.2. ISOQOL. |
| 149 | Lapin, Thompson, et al. (2018) Patient- versus proxy-response on global health scales: no meaningful difference. 2097. ISOQOL. |
| 150 | Lapin. (2024) Health-related quality of life for patients with post-acute COVID-19 syndrome: identification of symptom clusters and predictors of long-term outcomes. ISOQOL. |
| 151 | Lee et al. (2024) Crosswalk between PROMIS computer adaptive tests and numerical rating scales in cancer patients: Anxiety, Depression, Pain interference, Physical function. ISOQOL. |
| 152 | Lee M., & Nyman ( 2023) The effect of implementing shorter test length with PROMIS computer adaptive testing on score precision. PROMIS Health Organization Conference. |
| 153 | Leskó E 2024 Investigating how the QOL of oral cance patients is affeced by surgery using PROMIS. PROMIS Health Organization Conference. |
| 154 | Lindsey & McKelvey (2023) Identifying and reducing surgeon barriers and challenges in addressing mental health with patients. PROMIS Health Organization Conference. |
| 155 | Ling, Reynolds, et al. (2019) Adolescent Idiopathic Scoliosis patients with pain have lower pre- and post-operative Patient Reported Outcomes Measurement Information System (PROMIS) domain scores compared to their non-painful peers. O51. PROMIS Health Organization Conference. |
| 156 | Lingman, Chaplin, Nilsson, & Gustafsson. (2022) PRO, one key to information driven care in the Region of Halland, Sweden. JPRO, 7(1): P48 PROMIS Health Organization Conference. |
| 157 | MacDonald, Houck, & Baumhauer. (2017) Reading the future: Predicting who will benefit from bunion surgery. 31. PROMIS Health Organization Conference. |
| 158 | MacDonald, Houck, & Baumhauer. (2017) The road to recover for bunion surgery: Data analytic plots to target patient progress. P28. Health Measures User Conference. |
| 159 | MacDonald, Houck, & Baumhauer. (2017) The road to recovery for bunion surgery: Data analytic plots to target patient progress. 29. PROMIS Health Organization Conference. |
| 160 | MacDonald, Houck, et al. (2017) Trends in PROMIS scores in the early post-operative period following lateral ankle ligament reconstructive techniques. P28. Health Measures User Conference. |
| 161 | Makhni, Davis, Charters, ... Parsons. (2020) Design, development, and implementation of an integrated and automated patient repoted outcome measure platform through a native electronic health record: Results from the first 2,000 ambulatory encounters and 8,400 questionnaires administered. JPRO, 4(S2):137-P. PROMIS Health Organization Conference. |
| 162 | Manni, Cleveland, Whittaker, Zhang, Black, Haakenstad, ... McCleary. (2022) Evaluating systematic implementation of PROMIS-10 in ambulatory oncology: REALLS analysis of 10,691 patient responses. JPRO, 7(1): O55. PROMIS Health Organization Conference. |
| 163 | Marrache, Puvanesarah, et al. (2021) Persistent sleep disturbance after spine surgery is associated with failure to achieve maningful improvements in pain and health-related quality of life. B201.7. ISOQOL. |
| 164 | Martin, Brodke, et al. (2020) Association between the Surgical Invasiveness Index and patient-reported outcomes after lumbar spine surgery. 3141. ISOQOL. |
| 165 | McCleary, Sisodia, & Burnside. (2022) Realizing the promise of PROMIS: insights for one million responses. JPRO, 7(1): P47. PROMIS Health Organization Conference. |
| 166 | McCoy, Perry, et al. (2020) Leveraging EPIC to collect PROMIS CAT scores for orthopedic surgery patients to creat eblueprint for system-wide expansion. 111.1. ISOQOL. |
| 167 | McGuire, Yap, et al. (2019) Planning for a multicenter longitudinal pediatric implementation of PROMIS standard of care data collection. P41. Health Measures User Conference. |
| 168 | McIntyre & Baumhauer. (2018) PROgram management for ongoing PRO collections in a clinical setting. P056. PROMIS Health Organization Conference. |
| 169 | McKelvey, Dornan, Lindsey. (2022) Depression and suicide screening in orthopaedic clinics: balancing patient survey-response burden with best practices. MPRO, 7(1): O37. PROMIS Health Organization Conference. |
| 170 | Milewski, M., Coene, R., Cook, D., Kocher, M., Kramer, D., Micheli, L., Yen, Y. M., Christino, M., & Tarchala, M. (2023). Poster 347: PSYCHOLOGICAL READINESS AFTER REVISION ACL RECONSTRUCTION: A ROAR STUDY. Orthopaedic Journal of Sports Medicine, 11(7 suppl3), 2325967123S00313. https://doi.org/10.1177/2325967123S00313 |
| 171 | Millar, Manske, James. (2020) Is PROMIS a useful outcomes tool for children with Arthrogryposis? JPRO 4(2):146-P. PROMIS Health Organization Conference. |
| 172 | Mitten, Baumhauer, et al. (2016) Incidence of depressive symptoms in orthopaedic practice. Symposium 6. ISOQOL. |
| 173 | Muilekom et al. (2024) The Amsterdam PROM implementation strategy: policy and pathway. ISOQOL. |
| 174 | MyIntyre, Baumhauer, et al. (2016) Developing effective and efficient system to collect patient reported outcomes using the PROMIS in a clinical setting. Symposium 6. ISOQOL. |
| 175 | MyIntyre, Fear, & Hudy. (2019) Survey delivery windows to reduce respondent burden in clinical PRO collections. P40. PROMIS Health Organization Conference. |
| 176 | MyIntyre, Houck, et al. (2016) Reliability of PROMIS Physical Function CAT, Pain Interference CAT, and Depression CAT scores in patients with hip intra-articular pathology before and after arthroscopy. 1. PROMIS Health Organization Conference. |
| 177 | Nada Al-Antary et al. Assessment of patient reported outcomes (PROs) completion patterns in patients with cancer: Examining real-world data.. J Clin Oncol 41, 6609-6609(2023). DOI:10.1200/JCO.2023.41.16_suppl.6609 |
| 178 | Nelson, Burton, et al. (2019) Sociotechnical evaluation of PROMIS patient-reported outcome (PRO) measures in the Northwestern Memorial Hospital urology clinic. P64. Health Measures User Conference. |
| 179 | Neumayr, Treadwell, et al. (2017) Documenting sickle cell disease health realted quality of life (HRQOL) in the electronic medical record. P30. Health Measures User Conference. |
| 180 | Ng D.Q. et al (2023) Predicting symptom trajectories among cancer patients receiving treatment using machine learning: A PROMISing feasibility study. PROMIS Health Organization Conference. |
| 181 | Nyman M et al2024) Correlation of actual MET performance to DASI and PROMIS-PF patient report. PROMIS Health Organization Conference. |
| 182 | Nyman, Wolff, et al. (2019) Feasibility of using PROMIS-CAT to capture patient reported outcomes in neurosurgery outpatient setting. O44 PROMIS Health Organization Conference. |
| 183 | Paddock, Koenig, et al. (2017) Outcomes between the Depuy Sigma and Attune for total knee arthroplasty; does the implant matter? 44. PROMIS Health Organization Conference. |
| 184 | Papuga, Maloney, et al. (2016) The use of PROMIS CAT physical function pre-surgical scores to predict recovery from ACL reconstruction surgery. Symposium 6. ISOQOL. |
| 185 | Papuga, Maloney, et al. (2016)The use of PROMIS CAT post-surgical scores in assessing "value" in ACL reconstruction surgery. 38. PROMIS Health Organization Conference. |
| 186 | Patterson, Orvets, et al. (2017) Preoperative depression and anxiety predicts self-reported shoulder function in patients with symptomatic rotator cuff tears. 70. PROMIS Health Organization Conference. |
| 187 | Patterson, Orvets, et al. (2017) Validation of the Patient Reported Outcomes Measurement Information System (PROMIS) instruments in patients with rotator cuff diease. 71. PROMIS Health Organization Conference. |
| 188 | Peipert, Waterman, et al. (2019) Comparison of health-related quality of life among kidney and liver transplant patients using the PROMIS global health scale. O48. PROMIS Health Organization Conference. |
| 189 | Pennings J.S. et al (2023) PF and PI change scores associated with ODI, NDI, and satisfaction MCIDs after spine surgery. PROMIS Health Organization Conference. |
| 190 | Pennings, Bley, Coronado, & Archer. (2022) Relationship between preoperative expectations and physical function scores at 12 months after cervical spine surgery. JPRO, 7(1): P88. PROMIS Health Organization Conference. |
| 191 | Pennings, Coronado, Master, & Archer. (2021) Patient factors associated with improvemens in PROMIS Physical Function and Pain Interference after spine surgery. JPRO 5(1): O72. PROMIS Health Organization Conference. |
| 192 | Perry, Garcia, Kallen, Smith, Metzger, Cahue, Lancki, & Cella. (2022) Clinical implementation and validation of PROMIS 2-item short forms for routine symptom monitoring in oncology. JPRO, 7(1): O46. PROMIS Health Organization Conference. |
| 193 | Petroski, Kaczmarke, ... Gudesblatt. (2021) Multiple Sclerosis: the relationship of cognitive impairment and self-efficacy of medication management. JPRO 5(1): P46. PROMIS Health Organization Conference. |
| 194 | Philbrook, Jacobson, et al. (2019) Does health assessment using PROMIS scales enhance clinical decision making for patients attending physical therapy? P50. PROMIS Health Organization Conference. |
| 195 | Philip, Alimena, ... Sisodia. (2020) Patient reported outcomes after risk-reducing gynecologic surgery for hereditary breast and ovarian cancer cyndromes. JPRO, 4(S2):156-P. PROMIS Health Organization Conference. |
| 196 | Pugely, Gao, et al. (2017)The validity of PROMIS instruments among individuals with adult spinal deformity. 48. PROMIS Health Organization Conference. |
| 197 | Reynolds, Ling, et al. (2019) The importance of self-image for patients with adolescent idopathic scoliosis: limitations of existing PROMIS domains. P33. PROMIS Health Organization Conference. |
| 198 | Riggs, Madigan, et al. (2017) Feasibility of home health care patients' self-administration of the PROMIS global health survey. P34. Health Measures User Conference. |
| 199 | Rivera, Lacomis, Thirumala, & Hanmer. (2021) PROMIS validation in Myasthenia Gravis population. JPRO 5(1): P28. PROMIS Health Organization Conference. |
| 200 | Rothrock et al. (2018) Evaluation of the implementation of PROMIS CAT batteries for total join arthroplasty in an electronic health record. 108.3. ISOQOL. |
| 201 | Rubert, McIntyre, et al. (2018) Standard of care PRO collection across a healthcare system. S9. ISOQOL. |
| 202 | Ruebeck, Baumhauer, & Anandarajah. (2017) Impact of fibromyalgia (FMS) on PROMIS t-scores in patients with theumatoid arthritis (RA). 34. PROMIS Health Organization Conference. |
| 203 | S Shenoy, J Debroff, J Lacoste, E Giselbrecht, L Keefer, S Y Wong, P444 Active perianal Crohn’s disease negatively impacts patients’ quality of life, psychosocial wellbeing and is associated with barriers related to social determinants of health, Journal of Crohn's and Colitis, Volume 18, Issue Supplement_1, January 2024, Pages i898–i899, https://doi.org/10.1093/ecco-jcc/jjad212.0574 |
| 204 | Sachiv Chakravarti, Cathleen Kuo, Foad Kazemi, Atharv Oak, Calixto-Hope Lucas, Victoria Croog, David Kamson, Karisa Schreck, Matthias Holdhoff, Chetan Bettegowda, Debraj Mukherjee, QOL-15. PATIENT-REPORTED PHYSICAL HEALTH-RELATED QUALITY OF LIFE PREDICTS SHORT-TERM POSTOPERATIVE OUTCOMES IN A HETEROGENEOUS COHORT OF BRAIN TUMOR PATIENTS, Neuro-Oncology, Volume 26, Issue Supplement_8, November 2024, Page viii265, https://doi.org/10.1093/neuonc/noae165.1050 |
| 205 | Samudio A et al (2024) Frequency of common symptoms immediately after kidney and liver transplantation. PROMIS Health Organization Conference. |
| 206 | Schloesser. (2019) Implementation and utilization of patient-reported outcomes at the behavioral health point-of-care. P16. Health Measures User Conference. |
| 207 | Schmitt, Springsted, Ford, ... Nyman. (2021) A patient-centric PROMIS care continuum: tools to encourage patient and provider use of PROMIS data. JPRO 5(1): O5. PROMIS Health Organization Conference. |
| 208 | Schuetz, Fox, et al. (2019) An evidence-based approach to implementing the Patient Reported Outcomes Measurement Information System in a Bolivian public cancer center. P2. Health Measures User Conference. |
| 209 | Secrest, Taliercio, et al. (2020) Patient-reported outcomes can identify skin flaring in autoimmune blistering diseases. B203.4. ISOQOL. |
| 210 | Sethi, Kaczmarek, ... Gudesblatt. (2021) Parkinson's Disease psychosocial illness impact: relation to patient reported anxiety, depression, & sleep disturbance. JPRO 5(1): P44. PROMIS Health Organization Conference. |
| 211 | Shah, Scordo, et al. (2021) Successful incorporation of electronic patient-reported outcomes using PROMIS-29 into standard of care for lymphona patients receiving CD19-directed chimeric antigen receptor T (CAR T) cell therapy. 3039. ISOQOL. |
| 212 | Sharma H et al (2024) Implementing value-based care through an orthopedic integrated practice unit: Improved outcomes and reduced cost. PROMIS Health Organization Conference. |
| 213 | Shoghi, Balgey, & James. (2020) PROMIS scores for children with Unilateral Congenital Below Elbow Deficiency (UCBED). JRPO 4(S2):162-P. PROMIS Health Organization Conference. |
| 214 | Siembida, Corrigan, et al. The burden of radiation therapy in central nervous system (CNS) tumors: A descriptive analysis of health-realted quality of life in adolescent and young adult CNS cancer survivors. 104.5. ISOQOL. |
| 215 | Sikorskii A. et al. (2023) Patient-reported outcome thresholds for unscheduled health services in patients with cancer. PROMIS Health Organization Conference. |
| 216 | Sirois, Chiarlitti, et al. (2017) "I just want my life back": reducing fatigue and improving function are critical tragets for improving particiation and HRQL in rheumatoid arthritis. 1084. ISOQOL. |
| 217 | Skolasky, Li, et al. (2015) Improving patient centered care: implemtnation of PROMIS in a surgical spine practice. 2044. ISOQOL. |
| 218 | Smolyak, Jones, Ketonis. (2021) Presenting PROMIS scores predict treatment selection for De Quervain's Tenosynovitis. JPRO 5(1): P18. PROMIS Health Organization Conference. |
| 219 | Sokas, Cooper, et al. (2020) Associations between pre-operative social support and social functinoing and post-operative quality of life in patients undergoing radical cystectomy for bladder cancer. 3052. ISOQOL. |
| 220 | Spraggs-Hughes, Guattery, & Calfee. (2018) Clinical application and usage of patient reported outcome measures in an orthopaedic outpatient setting. P065. PROMIS Health Organization Conference. |
| 221 | Spraggs-Hughes, Guattery, & Calfee.(2018) Research applications of patient reported outcome measures in an academic orthopaedic surgery department. P066. PROMIS Health Organization Conference. |
| 222 | Spraggs-Hughes, Guattery, et al. (2018) Clinical application and usage of patient reported outcome measures in an orthopaedic outpatient setting. 1072. ISOQOL. |
| 223 | Spraggs-Hughes, Ricci, et al. (2016) Insurance status and patient outcomes in the orthopaedic outpatient setting. 57. PROMIS Health Organization Conference. |
| 224 | Spraggs-Hughes, Ricci, et al. (2016) PROMIS physical function, pain interference, and depression: Assessing outcomes in an orthopaedic trauma setting. 58. PROMIS Health Organization Conference. |
| 225 | Staples, Beran-Maryott, et al. (2019) Optimizing outcome collection in a student-run clinic: implementation of an electronically administered outcome measure. 1026. ISOQOL. |
| 226 | Steinbeck et al. (2023) Using electronic patient-reported outcome-monitoring to improve recovery in routine orthopedic care: results of a randomized controlled trial. ISOQOL. |
| 227 | Strong, Baumhauer, et al. (2017) Early PROMIS scores 6 weeks following total hip arthroplasty (THA) can identify patients who are unlikely to demonstrate improvement at greater than one year follow-up. 54. PROMIS Health Organization Conference. |
| 228 | Strong, Baumhauer, et al. (2017) Early PROMIS scores 6 weeks following total knee arthroplasty (TKA) can identify patients who are unlikely to demonstrate improvement at greater than one year follow-up. 55. PROMIS Health Organization Conference. |
| 229 | Strong, Baumhauer, et al. (2017) PROMIS scores following total hip arthroplasty demonstrate continued improvement at greater than one year. 56. PROMIS Health Organization Conference. |
| 230 | Strong, Baumhauer, et al. (2017) PROMIS scores following total knee arthroplasty demonstrate no further improvement between 6 months post-operatively to greater than 1 year post-operative. 58. PROMIS Health Organization Conference. |
| 231 | Strong, Maloney, et al. (2017) Psychometric evaluation of the PROMIS Physical Function CAT, PROMIS Pain Interference CAT, PROMIS Depression CAT, and ASES for a variety of shoulder diagnoses. 57. PROMIS Health Organization Conference. |
| 232 | Strong, Strong, et al. (2017) Comparison of single-level and multilevel transoraminal epidural steroid injections utilizing PROMIS outcomes data. P37. Health Measures User Conference. |
| 233 | Strong, Strong, et al. (2017) Establishing PROMIS minimal clinically important difference (MCID) values after single-level and multilevel transforaminal epidural steriod injections. P37. Health Measures User Conference. |
| 234 | Stukenborg, Blackhall, et al. (2015) Collection and use of PROMIS measures for palliative care cancer patients. PROMIS Health Organization Conference. |
| 235 | Taylor, Meta, et al. (2017) Hip arthroscopy from the PROMIS perspective: An analysis of prospectively collected patient reported outcomes data. 87. PROMIS Health Organization Conference. |
| 236 | Thackeray A, et al (2024)Measuring health profiles among physical therapy patients using a global measure. PROMIS Health Organization Conference. |
| 237 | Tierney, Kaczmarek, ... Gudesblatt. (2021) Parkinson's Disease and medication management: the relationship of cognitive ability - perception vs performance. JPRO 5(1): O43. PROMIS Health Organization Conference. |
| 238 | Tinsley. (2021) Patient reported measures- driving system transformation through measuring what matters to our patients in New South Wales. JPRO 5(1): P2. PROMIS Health Organization Conference. |
| 239 | Titmuss, Pan, et al. (2017) Implementing PROMIS as standard of care in a high surgical volume, nationally leading orthopedic hospital. 78. PROMIS Health Organization Conference. |
| 240 | Torchia, Keeney, et al. (2017) The relationship of PROMIS-10 physical function change and satisfaction following total knee arthroplasty. 62. PROMIS Health Organization Conference. |
| 241 | Tramer, Franovic, ... Makhni. (2020) Role of pre-operative PROMIS scores in predicting post-operative outcomes and likelihood of achieiving MCID following arthroscopic rotator cuff repair. JPRO, 4(S2):138-P. PROMIS Health Organization Conference. |
| 242 | Utsch M et al (2024) PROMIS GH-10 shows significant pre-post effects on physical and mental health Swiss inpatient rehabilitation. PROMIS Health Organization Conference. |
| 243 | Valencia, Jo, & Kim. (2021) Femoral head deformity correlates with physical and mental health measures in healed Stage Legg-Calve-Perthes disease. JPRO 5(1): P36. PROMIS Health Organization Conference. |
| 244 | van Vree, Volker, et al. (2018) Is PROMIS promising in rehabilitation? A pilot study in regular care. O068. PROMIS Health Organization Conference. |
| 245 | Vasalos, Santer, et al. (2018) Can patient reported outcomes guide therapy eneds in foot and ankle patients? P048. PROMIS Health Organization Conference. |
| 246 | Verma & Navarro. (2015) Integrating patient reported outcomes assessment in advanced liver disease. PROMIS Health Organization Conference. |
| 247 | Wahl, Gross, et al. (2015) Comparing PROMIS physical function short form 10a (PF-10a) to a clinical disease activity measure in rheumatoid arthritis patients. PROMIS Health Organization Conference. |
| 248 | Walbert, Lonni Schultz, James Matthew Snyder, Josh D Mohn, QOL-12. ELECTRONIC ASSESSMENT OF QUALITY OF LIFE IN GLIOBLASTOMA PATIENTS USING THE EORTC-QLQ-C30/ EORTC-BN20 AND THE PROMIS SYSTEM IN CLINICAL PRACTICE, Neuro-Oncology, Volume 26, Issue Supplement_8, November 2024, Page viii264, https://doi.org/10.1093/neuonc/noae165.1047 |
| 249 | Waldman, Houck, et al. (2019) Are global pain interference, physical function and depression important problems in patients with diabetic foot ulcers? P7. PROMIS Health Organization Conference. |
| 250 | Wasim, Gupta, .... Mucsi. (2022) Two-step screening for anxiety symptoms in kidney transplant recipients. JPRO, 7(1): P72. PROMIS Health Organization Conference. |
| 251 | Waters, Ries, et al. (2018) Collection of PROMIS measures using a digital platform Interim results from a feasibility study. P069. PROMIS Health Organization Conference. |
| 252 | Weeks, Hess, et al. (2017) Maternal mental health and patient-reported outcomes: identifying at-risk pregnant and postpartum patients. 3047. ISOQOL. |
| 253 | Wenzel, Tucker, et al. (2018) PROMIS emotional distress scores as cues for action. P070. PROMIS Health Organization Conference. |
| 254 | Willard et al (2024) Quantitative and qualitative assessment of clinician experience with PROMIS Global data collection. PROMIS Health Organization Conference. |
| 255 | Wisniewski M. et al., A promis to sreen for anxiety and depression in pediatric heart failure..Cambridge University Press 35, S2(2025). doi:10.1017/S1047951125000708 suppl S1 |
| 256 | Wong, Ling, et al. (2019) Assessing use of PROMIS outcomes in pediatric neuromuscular scoliosis patients. JPRO 3(S1):P3. PROMIS Health Organization Conference. |
| 257 | Wright, Beleckas, et al. (2018) Social deprivation's impact on physical and mental health in orthopedic patients. P071. PROMIS Health Organization Conference. |
| 258 | Yantsis, Markova, ... Mucsi. (2022) Assessing pain among solid organ transplant recipients using PROMIS tools. JPRO 7(1): P80. PROMIS Health Organization Conference. |
| 259 | Zegman, Elias, et al. (2016) Using a multi-modal, longitudinal collection strategy to collect PROMIS scales across a healthcare delivery system. 31. PROMIS Health Organization Conference. |
| 260 | Zeng et al. (2023) Latent class analysis reveals unmet social needs subgroups with differential outcomes within a large healthcare system. ISOQOL. |
| 261 | Ziarati, Moore, et al. (2019) Do patient reported outcomes correlate with clinical findings in patients undergoing total knee arthroplasty? O70. PROMIS Health Organization Conference. |
